# Abstract representations of events arise from mental errors in learning and memory

**DOI:** 10.1038/s41467-020-15146-7

**Published:** 2020-05-08

**Authors:** Christopher W. Lynn, Ari E. Kahn, Nathaniel Nyema, Danielle S. Bassett

**Affiliations:** 1 Department of Physics & Astronomy, College of Arts & Sciences, University of Pennsylvania, Philadelphia, PA 19104 USA; 2 Department of Neuroscience, Perelman School of Medicine, University of Pennsylvania, Philadelphia, PA 19104 USA; 3 Department of Bioengineering, School of Engineering & Applied Science, University of Pennsylvania, Philadelphia, PA 19104 USA; 4 Department of Electrical & Systems Engineering, School of Engineering & Applied Science, University of Pennsylvania, Philadelphia, PA 19104 USA; 5 Department of Neurology, Perelman School of Medicine, University of Pennsylvania, Philadelphia, PA 19104 USA; 6 Department of Psychiatry, Perelman School of Medicine, University of Pennsylvania, Philadelphia, PA 19104 USA; 7 Santa Fe Institute, Santa Fe, NM 87501 USA

**Keywords:** Human behaviour, Complex networks, Statistical physics

## Abstract

Humans are adept at uncovering abstract associations in the world around them, yet the underlying mechanisms remain poorly understood. Intuitively, learning the higher-order structure of statistical relationships should involve complex mental processes. Here we propose an alternative perspective: that higher-order associations instead arise from natural errors in learning and memory. Using the free energy principle, which bridges information theory and Bayesian inference, we derive a maximum entropy model of people’s internal representations of the transitions between stimuli. Importantly, our model (i) affords a concise analytic form, (ii) qualitatively explains the effects of transition network structure on human expectations, and (iii) quantitatively predicts human reaction times in probabilistic sequential motor tasks. Together, these results suggest that mental errors influence our abstract representations of the world in significant and predictable ways, with direct implications for the study and design of optimally learnable information sources.

## Abstract (web summary)

Humans can easily uncover abstract associations. Here, the authors propose that higher-order associations arise from natural errors in learning and memory. They suggest that mental errors influence the humans’ representation of the world in significant and predictable ways.

## Introduction

Our experience of the world is punctuated in time by discrete events, all connected by an architecture of hidden forces and causes. In order to form expectations about the future, one of the brain’s primary functions is to infer the statistical structure underlying past experiences^1–3^. In fact, even within the first year of life, infants reliably detect the frequency with which one phoneme follows another in spoken language^4^. By the time we reach adulthood, uncovering statistical relationships between items and events enables us to perform abstract reasoning^5^, identify visual patterns^6^, produce language^7^, develop social intuition^8^, and segment continuous streams of data into self-similar parcels^9^. Notably, each of these functions requires the brain to identify statistical regularities across a range of scales. It has long been known that people are sensitive to differences in individual transition probabilities such as those between words or concepts^4,6^. In addition, mounting evidence suggests that humans can also infer abstract (or higher-order) statistical structures, including hierarchical patterns within sequences of stimuli^10^, temporal regularities on both global and local scales^11^, abstract concepts within webs of semantic relationships^12^, and general features of sparse data^13^.

To study this wide range of statistical structures in a unified framework, scientists have increasingly employed the language of network science^14^, wherein stimuli or states are conceptualized as nodes in a graph with edges or connections representing possible transitions between them. In this way, a sequence of stimuli often reflects a random walk along an underlying transition network^15–17^, and we can begin to ask which network features give rise to variations in human learning and behavior. This perspective has been particularly useful, for example, in the study of artificial grammars^18^, wherein human subjects are tasked with inferring the grammar rules (i.e., the network of transitions between letters and words) underlying a fabricated language^19^. Complementary research in statistical learning has demonstrated that modules (i.e., communities of densely connected nodes) within transition networks are reflected in brain imaging data^20^ and give rise to stark shifts in human reaction times^21^. Together, these efforts have culminated in a general realization that people’s internal representations of a transition structure are strongly influenced by its higher-order organization^22,23^. But how does the brain learn these abstract network features? Does the inference of higher-order relationships require sophisticated hierarchical learning algorithms? Or instead, do natural errors in cognition yield a “blurry” representation, making the coarse-grained architecture readily apparent?

To answer these questions, here we propose a single driving hypothesis: that when building models of the world, the brain is finely tuned to maximize accuracy while simultaneously minimizing computational complexity. Generally, this assumption stems from a rich history exploring the trade-off between brain function and computational cost^24,25^, from sparse coding principles at the neuronal level^26^ to the competition between information integration and segregation at the whole-brain level^27^ to the notion of exploration versus exploitation^28^ and the speed-accuracy trade-off^29^ at the behavioral level. To formalize our hypothesis, we employ the free energy principle^30^, which has become increasingly utilized to investigate constraints on cognitive functioning^31^ and explain how biological systems maintain efficient representations of the world around them^32^. Despite this thorough treatment of the accuracy-complexity trade-off in neuroscience and psychology, the prevailing intuition in statistical learning maintains that the brain is either optimized to perform Bayesian inference^12,13^, which is inherently error free, or hierarchical learning^10,11,16,18^, which typically entails increased rather than decreased computational complexity.

Here, we show that the competition between accuracy and computational complexity leads to a maximum entropy (or minimum complexity) model of people’s internal representations of events^30,33^. As we decrease the complexity of our model, allowing mental errors to take effect, higher-order features of the transition network organically come into focus while the fine-scale structure fades away, thus providing a concise mechanism explaining how people infer abstract statistical relationships. To a broad audience, our model provides an accessible mapping from transition networks to human behaviors, with particular relevance for the study and design of optimally learnable transition structures—either between words in spoken and written language^18,19,33^, notes in music^34^, or even concepts in classroom lectures^35^.

## Results

### Network effects on human expectations

**Fig. 1:**
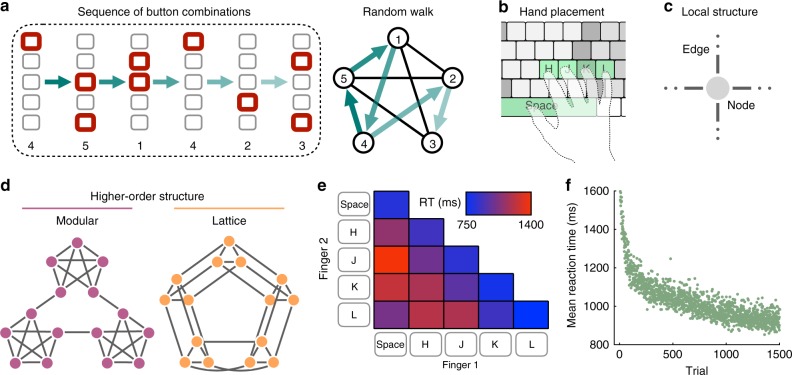
Subjects respond to sequences of stimuli drawn as random walks on an underlying transition graph. **a** Example sequence of visual stimuli (left) representing a random walk on an underlying transition network (right). **b** For each stimulus, subjects are asked to respond by pressing a combination of one or two buttons on a keyboard. **c** Each of the 15 possible button combinations corresponds to a node in the transition network. We only consider networks with nodes of uniform degree *k* = 4 and edges with uniform transition probability 0.25. **d** Subjects were asked to respond to sequences of 1500 such nodes drawn from two different transition architectures: a modular graph (left) and a lattice graph (right). **e** Average reaction times for the different button combinations, where the diagonal elements represent single-button presses and the off-diagonal elements represent two-button presses. **f** Average reaction times as a function of trial number, characterized by a steep drop-off in the first 500 trials followed by a gradual decline in the remaining 1000 trials. In **e** and **f**, averages are taken over responses during random walks on the modular and lattice graphs. Source data are provided as a Source Data file.

In the cognitive sciences, mounting evidence suggests that human expectations depend critically on the higher-order features of transition networks^15,16^. Here, we make this notion concrete with empirical evidence for higher-order network effects in a probabilistic sequential response task^22^. Specifically, we presented human subjects with sequences of stimuli on a computer screen, each stimulus depicting a row of five gray squares with one or two of the squares highlighted in red (Fig. 1a). In response to each stimulus, subjects were asked to press one or two computer keys mirroring the highlighted squares (Fig. 1b). Each of the 15 different stimuli represented a node in an underlying transition network, upon which a random walk stipulated the sequential order of stimuli (Fig. 1a). By measuring the speed with which a subject responded to each stimulus, we were able to infer their expectations about the transition structure: a fast reaction reflected a strongly anticipated transition, while a slow reaction reflected a weakly anticipated (or surprising) transition^1,2,22,36^.

While it has long been known that humans can detect differences in transition probabilities—for instance, rare transitions lead to sharp increases in reaction times^4,6^—more recently it has become clear that people’s expectations also reflect the higher-order architecture of transition networks^20–23,37^. To clearly study these higher-order effects without the confounding influence of variations in transition probabilities, here we only consider transition graphs with a uniform transition probability of 0.25 on each edge, thereby requiring nodes to have uniform degree *k* = 4 (Fig. 1c). Specifically, we consider two different graph topologies: a modular graph with three communities of five densely connected nodes and a lattice graph representing a 3 × 5 grid with periodic boundary conditions (Fig. 1d). As all transitions across both graphs have uniform probability, any systematic variations in behavior between different parts of a graph, or between the two graphs themselves, must stem from differences in the graphs’ higher-order modular or lattice structures.

**Fig. 2:**
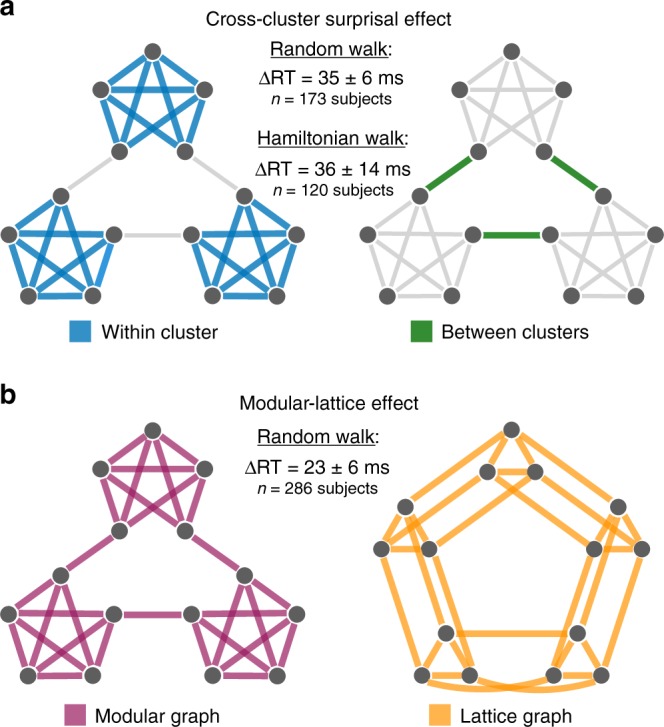
The effects of higher-order network structure on human reaction times. **a** Cross-cluster surprisal effect in the modular graph, defined by an average increase in reaction times for between-cluster transitions (right) relative to within-cluster transitions (left). We detect significant differences in reaction times for random walks (*p* < 0.001, *t* = 5.77, df = 1.61 × 10^5^) and Hamiltonian walks (*p* = 0.010, *t* = 2.59, df = 1.31 × 10^4^). For the mixed effects models used to estimate these effects, see Supplementary Tables 1 and 3. **b** Modular-lattice effect, characterized by an overall increase in reaction times in the lattice graph (right) relative to the modular graph (left). We detect a significant difference in reaction times for random walks (*p*  < 0.001, *t* = 3.95, df = 3.33 × 10^5^); see Supplementary Table 2 for the mixed effects model. Measurements were on independent subjects, statistical significance was computed using two-sided *F*-tests, and confidence intervals represent standard deviations. Source data are provided as a Source Data file.

Regressing out the dependence of reaction times on the different button combinations (Fig. 1e), the natural quickening of reactions with time^38^ (Fig. 1f), and the impact of stimulus recency (Methods), we identify two effects of higher-order network structure on subjects’ reactions. First, in the modular graph we find that reactions corresponding to within-cluster transitions are 35 ms faster than reactions to between-cluster transitions (*p* < 0.001, *F*-test; Supplementary Table 1), an effect known as the cross-cluster surprisal^22,37^ (Fig. 2a). Similarly, we find that people are more likely to respond correctly for within-cluster transitions than between-cluster transitions (Supplementary Table 8). Second, across all transitions within each network, we find that reactions in the modular graph are 23 ms faster than those in the lattice graph (*p* < 0.001, *F*-test; Supplementary Table 2), a phenomenon that we coin the modular-lattice effect (Fig. 2b).

Thus far, we have assumed that variations in human behavior stem from people’s internal expectations about the network structure. However, it is important to consider the possible confound of stimulus recency: the tendency for people to respond more quickly to stimuli that have appeared more recently^39,40^. To ensure that the observed network effects are not simply driven by recency, we performed a separate experiment that controlled for recency in the modular graph by presenting subjects with sequences of stimuli drawn according to Hamiltonian walks, which visit each node exactly once^20^. Within the Hamiltonian walks, we still detect a significant cross-cluster surprisal effect (Fig. 2a; Supplementary Tables 3–5). In addition, we controlled for recency in our initial random walk experiments by focusing on stimuli that previously appeared a specific number of trials in the past. Within these recency-controlled data, we find that both the cross-cluster surprisal and modular-lattice effects remain significant (Supplementary Figs. 2 and 3). Finally, for all of our analyses throughout the paper we regress out the dependence of reaction times on stimulus recency (Methods). Together, these results demonstrate that higher-order network effects on human behavior cannot be explained by recency alone.

In combination, our experimental observations indicate that people are sensitive to the higher-order architecture of transition networks. But how do people infer abstract features like community structure from sequences of stimuli? In what follows, we turn to the free energy principle to show that a possible answer lies in understanding the subtle role of mental errors.

### Network effects reveal errors in graph learning

As humans observe a sequence of stimuli or events, they construct an internal representation $\hat A$ of the transition structure, where $\hat A_{ij}$ represents the expected probability of transitioning from node *i* to node *j*. Given a running tally *n*_*ij*_ of the number of times each transition has occurred, one might naively expect that the human brain is optimized to learn the true transition structure as accurately as possible^41,42^. This common hypothesis is represented by the maximum likelihood estimate^43^, taking the simple form

1 $$\hat A_{ij}^{{\mathrm{MLE}}} = \frac{{n_{ij}}}{{\mathop {\sum}\nolimits_k {n_{ik}} }}.$$

To see that human behavior does not reflect maximum likelihood estimation, we note that Eq. (1) provides an unbiased estimate of the transition structure^43^; that is, the estimated transition probabilities in $\hat A^{{\mathrm{MLE}}}$ are evenly distributed about their true value 0.25, independent of the higher-order transition structure. Thus, the fact that people’s reaction times depend systematically on abstract features of the network marks a clear deviation from maximum likelihood estimation. To understand how higher-order network structure impacts people’s internal representations, we must delve deeper into the learning process itself.

Consider a sequence of nodes (*x*_1_, *x*_2_), where *x*_*t*_ ∈ {1, …, *N*} represents the node observed at time *t* and *N* is the size of the network (here *N* = 15 for all graphs). To update the maximum likelihood estimate of the transition structure at time *t* + 1, one increments the counts *n*_*ij*_ using the following recursive rule,

2 $$n_{ij}\left( {t + 1} \right) = n_{ij}\left( t \right) + \left[ {i = x_t} \right]\left[ {j = x_{t + 1}} \right],$$

where the Iverson bracket $\left[ \cdot \right] = 1$ if its argument is true and 0 otherwise. Importantly, we note that at each time *t* + 1, a person must recall the previous node that occurred at time *t*; in other words, they must associate a cause *x*_*t*_ to each effect *x*_*t*+1_ that they observe. Although maximum likelihood estimation requires perfect recollection of the previous node at each step, human errors in perception and recall are inevitable^44–46^. A more plausible scenario is that, when attempting to recall the node at time *t*, a person instead remembers the node at time *t*  −  Δ*t* with some decreasing probability *P*(Δ*t*), where Δ*t* ≥ 0. This memory distribution, in turn, generates an internal belief about which node occurred at time *t*,

3 $$B_t\left( i \right) = \mathop {\sum}\limits_{{\mathrm{\Delta }}t = 0}^{t - 1} {P\left( {{\mathrm{\Delta }}t} \right)\left[ {i = x_{t - {\mathrm{\Delta }}t}} \right]} .$$

Updating Eq. (2) accordingly, we arrive at a learning rule that accounts for natural errors in perception and recall,

4 $$\tilde n_{ij}\left( {t + 1} \right) = \tilde n_{ij}\left( t \right) + B_t\left( i \right)\left[ {j = x_{t + 1}} \right].$$

Using this revised counting rule, we can begin to form more realistic predictions about people’s internal estimates of the transition structure, $\hat A_{ij} = \tilde n_{ij}/\mathop {\sum}\nolimits_k {\tilde n_{ik}}$.

We remark that *P*(Δ*t*) does not represent the forgetting of past stimuli altogether; instead, it reflects the local shuffling of stimuli in time. If one were to forget past stimuli at some fixed rate – a process that is important for some cognitive functions^47^—this would merely introduce white noise into the maximum likelihood estimate $\hat A^{{\mathrm{MLE}}}$ (Supplementary Discussion). By contrast, we will see that, by shuffling the order of stimuli in time, people are able to gather information about the higher-order structure of the underlying transitions.

### Choosing a memory distribution: the free energy principle

In order to make predictions about people’s expectations, we must choose a particular mathematical form for the memory distribution *P*(Δ*t*). To do so, we begin with a single driving hypothesis: that the brain is finely tuned to (i) minimize errors and (ii) minimize computational complexity. Formally, we define the error of a recalled stimulus to be its distance in time from the desired stimulus (i.e., Δ*t*), such that the average error of a candidate distribution *Q*(Δ*t*) is given by $E\left( Q \right) = \mathop {\sum}\nolimits_{\Delta t} {Q\left( {{\mathrm{\Delta }}t} \right){\mathrm{\Delta }}t}$. By contrast, it might seem difficult to formalize the computational complexity associated with a distribution *Q*. Intuitively, we would like the complexity of *Q* to increase with increasing certainty. Moreover, as a first approximation we expect the complexity to be approximately additive such that the cost of storing two independent memories equals the costs of the two memories themselves. As famously shown by Shannon, these two criteria of monotonicity and additivity are sufficient to derive a quantitative definition of complexity^33^—namely, the negative entropy $- S\left( Q \right) = \mathop {\sum}\nolimits_{{\mathrm{\Delta }}t} {Q\left( {{\mathrm{\Delta }}t} \right){\mathrm{log}}Q\left( {{\mathrm{\Delta }}t} \right)}$.

Together, the total cost of a distribution *Q* is its free energy *F*(*Q*) = *βE*(*Q*) − *S*(*Q*), where *β* is the inverse temperature parameter, which quantifies the relative value that the brain places on accuracy versus efficiency^31^. In this way, our assumption about resource constraints in the brain leads to a particular form for *P*: it should be the distribution that minimizes *F*(*Q*), namely the Boltzmann distribution^30^

5 $$P\left( {{\mathrm{\Delta }}t} \right) = \frac{1}{Z}e^{ - \beta {\mathrm{\Delta }}t},$$

where *Z* is the normalizing constant (Methods). Free energy arguments similar to the one presented here have been used increasingly to formalize constraints on cognitive functions^31,32^, with applications from active inference^48^ and Bayesian learning under uncertainty^32^ to human action and perception with temporal or computational limitations^31,49,50^. Taken together, Eqs. (3)–(5) define our maximum entropy model of people’s internal transition estimates $\hat A$.

**Fig. 3:**
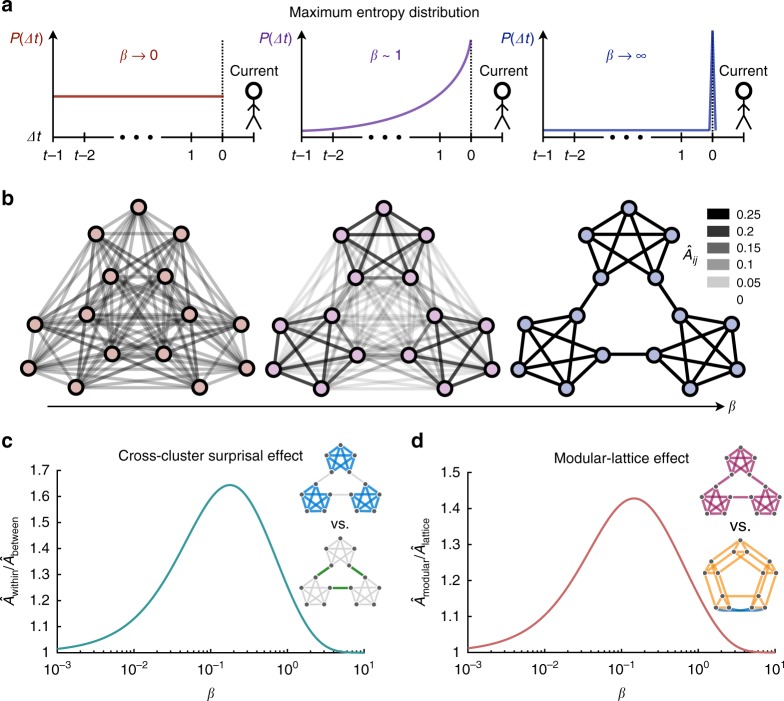
A maximum entropy model of transition probability estimates in humans. **a** Illustration of the maximum entropy distribution *P*(Δ*t*) representing the probability of recalling a stimulus Δ*t* time steps from the target stimulus (dashed line). In the limit *β* → 0, the distribution becomes uniform over all past stimuli (left). In the opposite limit *β* → ∞, the distribution becomes a delta function on the desired stimulus (right). For intermediate amounts of noise, the distribution drops off monotonically (center). **b** Resulting internal estimates $\hat A$ of the transition structure. For *β* → 0, the estimates become all-to-all, losing any resemblance to the true structure (left), while for *β* → ∞, the transition estimates become exact (right). At intermediate precision, the higher-order community structure organically comes into focus (center). **c**, **d** Predictions of the cross-cluster surprisal effect (**c**) and the modular-lattice effect (**d**) as functions of the inverse temperature *β*.

To gain an intuition for the model, we consider the infinite-time limit, such that the transition estimates become independent of the particular random walk chosen for analysis. Given a transition matrix *A*, one can show that the asymptotic estimates in our model are equivalent to an average over walks of various lengths, $\hat A = \mathop {\sum}\nolimits_{{\mathrm{\Delta }}t} {P\left( {{\mathrm{\Delta }}t} \right)A^{{\mathrm{\Delta }}t + 1}}$, which, in turn, can be fashioned into the following analytic expression,

6 $$\hat A = \left( {1 - e^{ - \beta }} \right)A\left( {I - e^{ - \beta }A} \right)^{ - 1},$$

where *I* is the identity matrix (Methods). The model contains a single free parameter *β*, which represents the precision of a person’s mental representation. In the limit *β* → ∞ (no mental errors), our model becomes equivalent to maximum likelihood estimation (Fig. 3a), and the asymptotic estimates $\hat A$ converge to the true transition structure *A* (Fig. 3b), as expected^51^. Conversely, in the limit *β* → 0 (overwhelming mental errors), the memory distribution *P*(Δ*t*) becomes uniform across all past nodes (Fig. 3a), and the mental representation $\hat A$ loses all resemblance to the true structure *A* (Fig. 3b).

Remarkably, for intermediate values of *β*, higher-order features of the transition network, such as communities of densely connected nodes, come into focus, while some of the fine-scale features, like the edges between communities, fade away (Fig. 3b). Applying Eq. (6) to the modular graph, we find that the average expected probability of within-community transitions reaches over 1.6 times the estimated probability of between-community transitions (Fig. 3c), thus explaining the cross-cluster surprisal effect^22,37^. Furthermore, we find that the average estimated transition probabilities in the modular graph reach over 1.4 times the estimated probabilities in the lattice graph (Fig. 3d), thereby predicting the modular-lattice effect. In addition to these higher-order effects, we find that the model also explains previously reported variations in human expectations at the level of individual nodes^4,6,22^ (Supplementary Fig. 1). Together, these results demonstrate that the maximum entropy model predicts the qualitative effects of network structure on human reaction times. But can we use the same ideas to quantitatively predict the behavior of particular individuals?

### Predicting the behavior of individual humans

To model the behavior of individual subjects, we relate the transition estimates in Eqs. (3)–(5) to predictions about people’s reaction times. Given a sequence of nodes *x*_1_, …, *x*_*t*−1_, we note that the reaction to the next node *x*_*t*_ is determined by the expected probability of transitioning from *x*_*t*−1_ to *x*_*t*_ calculated at time *t* − 1, which we denote by $a\left( t \right) = \hat A_{x_{t - 1},x_t}\left( {t - 1} \right)$. From this internal anticipation *a*(*t*), the simplest possible prediction $\hat r\left( t \right)$ for a person’s reaction time is given by the linear relationship^52^ $\hat r\left( t \right) = r_0 + r_1a\left( t \right)$, where the intercept *r*_0_ represents a person’s reaction time with zero anticipation and the slope *r*_1_ quantifies the strength of the relationship between a person’s reactions and their anticipation in our model^53^.

**Fig. 4:**
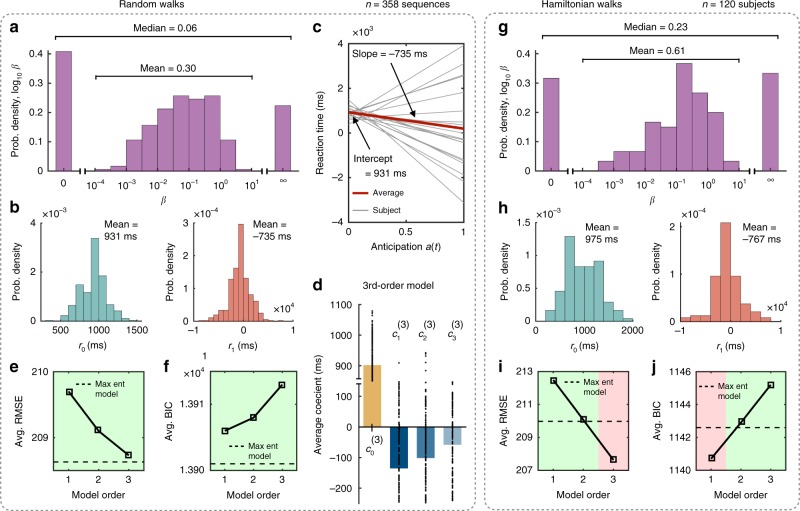
Predicting reaction times for individual subjects. **a**–**f** Estimated parameters and accuracy analysis for our maximum entropy model across 358 random walk sequences (across 286 subjects; Methods). **a** For the inverse temperature *β*, 40 sequences corresponded to the limit *β* → ∞, 73 corresponded to the limit *β* → 0. Among the remaining 245 sequences, the average value of *β* was 0.30. **b** Distributions of the intercept *r*_0_ (left) and slope *r*_1_ (right). **c** Predicted reaction time as a function of a subject’s internal anticipation. Gray lines indicate 20 randomly selected sequences, and the red line shows the average prediction over all sequences. **d** Linear parameters for the third-order competing model; data points represent individual sequences and bars represent averages. **e**, **f** Comparing the performance of our maximum entropy model with the hierarchy of competing models up to third-order. Root mean squared error (RMSE; **e**) and Bayesian information criterion (BIC; **f**) of our model averaged over all sequences (dashed lines) compared to the competing models (solid lines); our model provides the best description of the data across all models considered. **g**–**j** Estimated parameters and accuracy analysis for our maximum entropy model across all Hamiltonian walk sequences (120 subjects). **g** For the inverse temperature *β*, 20 subjects were best described as performing maximum likelihood estimation (*β* → ∞), 19 lacked any notion of the transition structure (*β* → 0), and the remaining 81 subjects had an average value of *β* = 0.61. **h** Distributions of the intercept *r*_0_ (left) and slope *r*_1_ (right). **i** Average RMSE of our model (dashed line) compared to that of the competing models (solid line); our model maintains higher accuracy than the competing hierarchy up to the second-order model. **j** Average BIC of the maximum entropy model (dashed line) compared to that of the competing models (solid line); our model provides a better description of the data than the second- or third-order models. Source data are provided as a Source Data file.

To estimate the parameters *β*, *r*_0_, and *r*_1_ that best describe a given individual, we minimize the root mean squared error (RMSE) between their predicted and observed reaction times after regressing out the dependencies on button combination, trial number, and recency (Fig. 1e, f; Methods). The distributions of the estimated parameters are shown in Fig. 4a–b for random walks and in Fig. 4g–h for Hamiltonian walks. Among the 358 random walk sequences in the modular and lattice graphs (across 286 subjects; Methods), 40 were best described as performing maximum likelihood estimation (*β* → ∞) and 73 seemed to lack any notion of the transition structure whatsoever (*β* → 0), while among the remaining 245 sequences, the average inverse temperature was *β* = 0.30. Meanwhile, among the 120 subjects that responded to Hamiltonian walk sequences, 81 appeared to have a non-trivial value of *β*, with an average of *β* = 0.61. Interestingly, these estimates of *β* roughly correspond to the values for which our model predicts the strongest network effects (Fig. 3c, d). In the following section, we will compare these values of *β*, which are estimated indirectly from people’s reaction times, with direct measurements of *β* in an independent memory experiment.

In addition to estimating *β*, we also wish to determine whether our model accurately describes individual behavior. Toward this end, we first note that the average slope *r*_1_ is large (−735 ms for random walks and −767 ms for Hamiltonian walks), suggesting that the transition estimates in our model *a*(*t*) are strongly predictive of human reaction times, and negative, confirming the intuition that increased anticipation yields decreased reaction times (Fig. 4b, h). To examine the accuracy of our model $\hat r$, we consider a hierarchy of competing models $\hat r^{\left( \ell \right)}$, which represent the hypothesis that humans learn explicit representations of the higher-order transition structure. In particular, we denote the $\ell{{\mathrm{th}}}$-order transition matrix by $\hat A_{ij}^{\left( \ell \right)} = n_{ij}^{\left( \ell \right)}/\mathop {\sum}\nolimits_k {n_{ik}^{\left( \ell \right)}}$, where $n_{ij}^{\left( \ell \right)}$ counts the number of observed transitions from node *i* to node *j* in $\ell$ steps. The model hierarchy takes into account increasingly higher-order transitions, such that the $\ell {{\mathrm{th}}}$-order model contains perfect information about transitions up to length $\ell$:

7 $$\hat r^{\left( 0 \right)}\left( t \right) =	 \ c_0^{\left( 0 \right)},\\ \hat r^{\left( 1 \right)}\left( t \right) =	 \ c_0^{\left( 1 \right)} + c_1^{\left( 1 \right)}a^{\left( 1 \right)}\left( t \right),\\ \quad \quad \quad 	\vdots \\ \hat r^{\left( \ell \right)}\left( t \right) =	 \ c_0^{\left( \ell \right)} + \mathop {\sum}\limits_{k = 1}^\ell {c_k^{\left( \ell \right)}a^{\left( k \right)}\left( t \right)} ,$$

where $a^{\left( k \right)}\left( t \right) = \hat A_{x_{t - 1},x_t}^{\left( k \right)}\left( {t - 1} \right)$. Each model $\hat r^{\left( \ell \right)}$ contains $\ell + 1$ parameters $c_0^{\left( \ell \right)},\; \ldots ,\;c_\ell ^{\left( \ell \right)}$, where $c_k^{\left( \ell \right)}$ quantifies the predictive power of the *k*th-order transition structure.

Intuitively, for each model $\hat r^{\left( \ell \right)}$, we expect $c_1^{\left( \ell \right)},c_2^{\left( \ell \right)}, \ldots$, to be negative, reflecting a decrease in reaction times due to increased anticipation, and decreasing in magnitude, such that higher-order transitions are progressively less predictive of people’s reaction times. Indeed, considering the third-order model $\hat r^{\left( 3 \right)}$ as an example, we find that progressively higher-order transitions are less predictive of human reactions (Fig. 4d). However, even the largest coefficient ($c_1^{\left( 3 \right)} = - 135\;{\mathrm{ms}}$) is much smaller than the slope in our maximum entropy model (*r*_1_ = −735 ms), indicating that the representation $\hat A$ is more strongly predictive of people’s reaction times than any of the explicit representations $\hat A^{\left( 1 \right)},\hat A^{\left( 2 \right)}$, …. Indeed, averaging over the random walk sequences, the maximum entropy model achieves higher accuracy than the first three orders of the competing model hierarchy (Fig. 4e)—this is despite the fact that the third-order model even contains one more parameter. To account for differences in the number of parameters, we additionally compare the average Bayesian information criterion (BIC) of our model with that of the competing models, finding that the maximum entropy model provides the best description of the data (Fig. 4f).

Similarly, averaging over the Hamiltonian walk sequences, the maximum entropy model provides more accurate predictions than the first two competing models (Fig. 4i) and provides a lower BIC than the second and third competing models (Fig. 4j). Notably, even in Hamiltonian walks, the maximum entropy model provides a better description of human reaction times than the second-order competing model, which has the same number of parameters. However, we remark that the first-order competing model has a lower BIC than the maximum entropy model (Fig. 4j), suggesting that humans may focus on first-order rather than higher-order statistics during Hamiltonian walks—an interesting direction for future research. On the whole, these results indicate that the free energy principle, and the resulting maximum entropy model, is consistently more effective at describing human reactions than the hypothesis that people learn explicit representations of the higher-order transition structure.

### Directly probing the memory distribution

Throughout our discussion, we have argued that errors in memory shape human representations in predictable ways, a perspective that has received increasing attention in recent years^47,54,55^. Although our framework explains specific aspects of human behavior, there exist alternative perspectives that might yield similar predictions. For example, one could imagine a Bayesian learner with a non-Markov prior that “integrates” the transition structure over time, even without sustaining errors in memory or learning. In addition, Eq. (6) resembles the successor representation in reinforcement learning^56,57^, which assumes that, rather than shuffling the order of past stimuli, humans are instead planning their responses multiple steps in advance (Supplementary Discussion). In order to distinguish our framework from these alternatives, here we provide direct evidence for precisely the types of mental errors predicted by our model.

In the construction and testing of our model, we have developed a series of predictions concerning the shape of the memory distribution *P*(Δ*t*), which, to recall, represents the probability of remembering the stimulus at time *t* − Δ*t* instead of the target stimulus at time *t*. We first assumed that *P*(Δ*t*) decreases monotonically. Second, to make quantitative predictions, we employed the free energy principle, leading to the prediction that *P* drops off exponentially quickly with Δ*t* (Eq. (5)). Finally, when fitting the model to individual subjects, we estimated an average inverse temperature *β* between 0.30 for random walks and 0.61 for Hamiltonian walks.

**Fig. 5:**
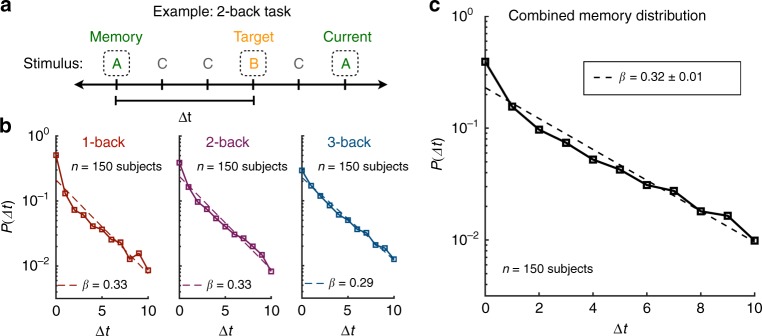
Measuring the memory distribution in an *n*-back experiment. **a** Example of the 2-back memory task. Subjects view a sequence of stimuli (letters) and respond to each stimulus indicating whether it matches the target stimulus from two trials before. For each positive response that the current stimulus matches the target, we measure Δ*t* by calculating the number of trials between the last instance of the current stimulus and the target. **b** Histograms of Δ*t* (i.e., measurements of the memory distribution *P*(Δ*t*)) across all subjects in the 1-, 2-, and 3-back tasks. Dashed lines indicate exponential fits to the observed distributions. The inverse temperature *β* is estimated for each task to be the negative slope of the exponential fit. **c** Memory distribution aggregated across the three *n*-back tasks. Dashed line indicates an exponential fit. We report a combined estimate of the inverse temperature *β* = 0.32 ± 0.01, where the standard deviation is estimated from 1000 bootstrap samples of the combined data. Measurements were on independent subjects. Source data are provided as a Source Data file.

To test these three predictions directly, we conducted a standard *n*-back memory experiment. Specifically, we presented subjects with sequences of letters on a screen, and they were asked to respond to each letter indicating whether or not it was the same as the letter that occurred *n* steps previously; for each subject, this process was repeated for the three conditions *n* = 1, 2, and 3. To measure the memory distribution *P*(Δ*t*), we considered all trials on which a subject responded positively that the current stimulus matched the target. For each such trial, we looked back to the last time that the subject did in fact observe the current stimulus and we recorded the distance (in trials) between this observation and the target (Fig. 5a). In this way, we were able to treat each positive response as a sample from the memory distribution *P*(Δ*t*).

The measurements of *P* for the 1-, 2-, and 3-back tasks are shown in Fig. 5b, and the combined measurement of *P* across all conditions is shown in Fig. 5c. Notably, the distributions decrease monotonically and maintain consistent exponential forms, even out to Δ*t* = 10 trials from the target stimulus, thereby providing direct evidence for the Boltzmann distribution (Eq. (5)). Moreover, fitting an exponential curve to each distribution, we can directly estimate the inverse temperature *β*. Remarkably, the value *β* = 0.32 ± 0.1 estimated from the combined distribution (Fig. 5c) falls within the range of values estimated from our reaction time experiments (Fig. 4a, g), nearly matching the average value *β* = 0.30 for random walk sequences (Fig. 4a).

To further strengthen the link between mental errors and people’s internal representations, we then asked subjects to perform the original serial response task (Fig. 1), and for each subject, we estimated *β* using the two methods described above: (i) directly measuring *β* in the *n*-back experiment, and (ii) indirectly estimating *β* in the serial response experiment. Comparing these two estimates across subjects, we find that they are significantly related with Spearman correlation *r*_s_ = 0.28 (*p* = 0.047, permutation test), while noting that we do not use the Pearson correlation because *β* is not normally distributed (Anderson–Darling test^58^, *p* < 0.001 for the serial response task and *p* = 0.013 for the *n*-back task). Together, these results demonstrate not only the existence of the particular form of mental errors predicted by our model—down to the specific value of *β*—but also the relationship between these mental errors and people’s internal estimates of the transition structure.

### Network structure guides reactions to novel transitions

Given a model of human behavior, it is ultimately interesting to make testable predictions. Thus far, in keeping with the majority of existing research^4,6,20–22,37^, we have focused on static transition graphs, wherein the probability *A*_*ij*_ of transitioning from state *i* to state *j* remains constant over time. However, the statistical structures governing human life are continually shifting^59,60^, and people are often forced to respond to rare or novel transitions^61,62^. Here we show that, when confronted with a novel transition—or a violation of the preexisting transition network—not only are people surprised, but the magnitude of their surprise depends critically on the topology of the underlying network.

**Fig. 6:**
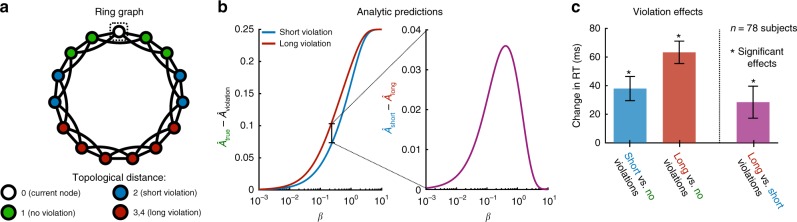
Network violations yield surprise that grows with topological distance. **a** Ring graph consisting of 15 nodes, where each node is connected to its nearest neighbors and next-nearest neighbors on the ring. Starting from the boxed node, a sequence can undergo a standard transition (green), a short violation of the transition structure (blue), or a long violation (red). **b** Our model predicts that subjects’ anticipations of both short (blue) and long (red) violations should be weaker than their anticipations of standard transitions (left). Furthermore, we predict that subjects’ anticipations of violations should decrease with increasing topological distance (right). **c** Average effects of network violations across 78 subjects, estimated using a mixed effects model (Supplementary Tables 10 and 11), with error bars indicating one standard deviation from the mean. We find that standard transitions yield quicker reactions than both short violations (*p* < 0.001, *t* = 4.50, df = 7.15 × 10^4^) and long violations (*p* < 0.001, *t* = 8.07, df = 7.15 × 10^4^). Moreover, topologically shorter violations induce faster reactions than long violations (*p* = 0.011, *t* = 2.54, df = 3.44 × 10^3^), thus confirming the predictions of our model. Measurements were on independent subjects, and statistical significance was computed using two-sided *F*-tests. Source data are provided as a Source Data file.

We consider a ring graph where each node is connected to its nearest and next-nearest neighbors (Fig. 6a). We asked subjects to respond to sequences of 1500 nodes drawn as random walks on the ring graph, but with 50 violations randomly interspersed. These violations were divided into two categories: short violations of topological distance two and long violations of topological distances three and four (Fig. 6a). Using maximum likelihood estimation (Eq. (1)) as a guide, one would naively expect people to be equally surprised by all violations—indeed, each violation has never been seen before. In contrast, our model predicts that that surprise should depend crucially on the topological distance of a violation in the underlying graph, with topologically longer violations inducing increased surprise over short violations (Fig. 6b).

In the data, we find that all violations give rise to sharp increases in reaction times relative to standard transitions (Fig. 6c; Supplementary Table 10), indicating that people are in fact learning the underlying transition structure. Moreover, we find that reaction times for long violations are 28 ms longer than those for short violations (*p* = 0.011, *F*-test; Fig. 6c; Supplementary Table 11). In addition, we confirm that the effects of network violations are not simply driven by stimulus recency (Supplementary Figs. 4 and 5). These observations suggest that people learn the topological distances between all nodes in the transition graph, not just those pairs for which a transition has already been observed^59–62^.

## Discussion

Daily life is filled with sequences of items that obey an underlying network architecture, from networks of word and note transitions in natural language and music to networks of abstract relationships in classroom lectures and literature^5–9^. How humans infer and internally represent these complex structures are questions of fundamental interest^10–13^. Recent experiments in statistical learning have established that human representations depend critically on the higher-order organization of probabilistic transitions, yet the underlying mechanisms remain poorly understood^20–23^.

Here we show that network effects on human behavior can be understood as stemming from mental errors in people’s estimates of the transition structure, while noting that future work should focus on disambiguating the role of recency^39,40^. We use the free energy principle to develop a model of human expectations that explicitly accounts for the brain’s natural tendency to minimize computational complexity—that is, to maximize entropy^31,32,49^. Indeed, the brain must balance the benefits of making accurate predictions against the computational costs associated with such predictions^24–29,50^. This competition between accuracy and efficiency induces errors in people’s internal representations, which, in turn, explains with notable accuracy an array of higher-order network phenomena observed in human experiments^20–23^. Importantly, our model admits a concise analytic form (Eq. (6)) and can be used to predict human behavior on a person-by-person basis (Fig. 4).

This work inspires directions for future research, particularly with regard to the study and design of optimally learnable network structures. Given the notion that densely connected communities help to mitigate the effects of mental errors on people’s internal representations, we anticipate that networks with high “learnability” will possess a hierarchical community structure^63^. Interestingly, such hierarchical organization has already been observed in a diverse range of real world networks, from knowledge and language graphs^64^ to social networks and the World Wide Web^65^. Could it be that these networks have evolved so as to facilitate accurate representations in the minds of the humans using and observing them? Questions such as this demonstrate the importance of having simple principled models of human representations and point to the promising future of this research endeavor.

## Methods

### Maximum entropy model and the infinite-sequence limit

Here we provide a more thorough derivation of our maximum entropy model of human expectations, with the goal of fostering intuition. Given a matrix of erroneous transition counts $\tilde n_{ij}$, our estimate of the transition structure is given by $\hat A_{ij} = \tilde n_{ij}/\mathop {\sum}\nolimits_k {\tilde n_{ik}}$. When observing a sequence of nodes *x*_1_, *x*_2_, …, in order to construct the counts $\tilde n_{ij}$, we assume that humans use the following recursive rule: $\tilde n_{ij}\left( {t + 1} \right) = \tilde n_{ij}\left( t \right) + B_t\left( i \right)\left[ {j = x_{t + 1}} \right]$, where *B*_*t*_(*i*) denotes the belief, or perceived probability, that node *i* occurred at the previous time *t*. This belief, in turn, can be written in terms of the probability *P*(Δ*t*) of accidentally recalling the node that occurred Δ*t* time steps from the desired node at time *t*: $B_t\left( i \right) = \mathop {\sum}\nolimits_{{\mathrm{\Delta }}t = 0}^{t - 1} {P\left( {{\mathrm{\Delta }}t} \right)\left[ {i = x_{t - {\mathrm{\Delta }}t}} \right]}$.

In order to make quantitative predictions about people’s estimates of a transition structure, we must choose a mathematical form for *P*(Δ*t*). To do so, we leverage the free energy principle^31^: When building mental models, the brain is finely tuned to simultaneously minimize errors and computational complexity. The average error associated with a candidate distribution *Q*(Δ*t*) is assumed to be the average distance in time of the recalled node from the target node, denoted $E\left( Q \right) = \mathop {\sum}\nolimits_{{\mathrm{\Delta }}t} {Q\left( {{\mathrm{\Delta }}t} \right){\mathrm{\Delta }}t}$. Furthermore, Shannon famously proved that the only suitable choice for the computational cost of a candidate distribution is its negative entropy^33^, denoted $- S\left( Q \right) = \mathop {\sum}\nolimits_{{\mathrm{\Delta }}t} {Q\left( {{\mathrm{\Delta }}t} \right){\mathrm{log}}Q\left( {{\mathrm{\Delta }}t} \right)}$. Taken together, the total cost associated with a distribution *Q*(Δ*t*) is given by the free energy *F*(*Q*) = *βE*(*Q*) − *S*(*Q*), where *β*, referred to as the inverse temperature, parameterizes the relative importance of minimizing errors versus computational costs. By minimizing *F* with respect to *Q*, we arrive at the Boltzmann distribution *p*(Δ*t*) = *e*^−*β*Δ*t*^/*Z*, where *Z* is the normalizing partition function^30^. We emphasize that this mathematical form for *P*(Δ*t*) followed directly from our free energy assumption about resource constraints in the brain.

To gain an analytic intuition for the model without referring to a particular random walk, we consider the limit of an infinitely long sequence of nodes. To begin, we consider a sequence *x*_1_, …, *x*_*T*_ of length *T*. At the end of this sequence, the counting matrix takes the form

8 $$\tilde n_{ij}\left( T \right) 	= \mathop {\sum}\limits_{t = 1}^{T - 1} {B_t\left( i \right)\left[ {j = x_{t + 1}} \right]} \\ 	= \mathop {\sum}\limits_{t = 1}^{{\it{T}} - 1} {\left( {\mathop {\sum}\limits_{{\mathrm{\Delta }}t = 0}^{t - 1} {P\left( {{\mathrm{\Delta }}t} \right)\left[ {i = x_{t - {\mathrm{\Delta }}t}} \right]} } \right)\left[ {j = x_{t + 1}} \right]}.$$

Dividing both sides by *T*, the right-hand side becomes a time average, which by the ergodic theorem converges to an expectation over the transition structure in the limit *T* → ∞,

9 $$\mathop {{{\mathrm{lim}}}}\limits_{T \to \infty } \frac{{\tilde n_{ij}\left( T \right)}}{T} = \mathop {\sum}\limits_{{\mathrm{\Delta }}t = 0}^\infty {P\left( {{\mathrm{\Delta }}t} \right)\left\langle {\left[ {i = x_{t - {\mathrm{\Delta }}t}} \right]\left[ {j = x_{t + 1}} \right]} \right\rangle } _A,$$

where $\left\langle \cdot \right\rangle _A$ denotes an expectation over random walks in *A*. We note that the expectation of an identity function is simply a probability, such that $\left\langle {\left[ {i = x_{t - {\mathrm{\Delta }}t}} \right]\left[ {j = x_{t + 1}} \right]} \right\rangle _A = p_i\left( {A^{{\mathrm{\Delta }}t + 1}} \right)_{ij}$, where *p*_*i*_ is the long-run probability of node *i* appearing in the sequence and $\left( {A^{{\mathrm{\Delta }}t + 1}} \right)_{ij}$ is the probability of randomly walking from node *i* to node *j* in Δ*t* + 1 steps. Putting these pieces together, we find that the expectation $\hat A$ converges to a concise mathematical form,

10 $$\mathop {{{\mathrm{lim}}}}\limits_{T \to \infty } \hat A_{ij}\left( T \right) 	= \mathop {{{\mathrm{lim}}}}\limits_{T \to \infty } \frac{{\tilde n_{ij}\left( T \right)}}{{\mathop {\sum}\nolimits_k {\tilde n_{ik}\left( T \right)} }}\\ 	= \frac{{p_i\mathop {\sum}\nolimits_{{\mathrm{\Delta }}t = 0}^\infty {P\left( {{\mathrm{\Delta }}t} \right)\left( {A^{{\mathrm{\Delta }}t + 1}} \right)_{ij}} }}{{p_i}}\\ 	= \mathop {\sum}\limits_{{\mathrm{\Delta }}t = 0}^\infty {P\left( {{\mathrm{\Delta }}t} \right)\left( {A^{{\mathrm{\Delta }}t + 1}} \right)_{ij}} .$$

Thus far, we have not appealed to our maximum entropy form for *P*(Δ*t*). It turns out that doing so allows us to write down an analytic expression for the long-time expectations $\hat A$ simply in terms of the transition structure *A* and the inverse temperature *β*. Noting that $Z = \mathop {\sum}\nolimits_{{\mathrm{\Delta }}t = 0}^\infty {e^{ - \beta {\mathrm{\Delta }}t} = 1/\left( {1 - e^{ - \beta }} \right)}$ and $\mathop {\sum}\nolimits_{{\mathrm{\Delta }}t = 0}^\infty {\left( {e^{ - \beta }A} \right)^{{\mathrm{\Delta }}t} = \left( {I - e^{ - \beta }A} \right)^{ - 1}}$, we have

11 $$\hat A 	= \mathop {\sum}\limits_{{\mathrm{\Delta }}t = 0}^\infty {P\left( {{\mathrm{\Delta }}t} \right)A^{{\mathrm{\Delta }}t + 1}} \\ 	= \frac{1}{Z}A\mathop {\sum}\limits_{{\mathrm{\Delta }}t = 0}^\infty {\left( {e^{ - \beta }A} \right)^{{\mathrm{\Delta }}t}} \\ 	= \left( {1 - e^{ - \beta }} \right)A\left( {I - e^{ - \beta }A} \right)^{ - 1}.$$

This simple formula for the representation $\hat A$ is the basis for all of our analytic predictions (Figs. 3c, d and 6b) and is closely related to notions of communicability in complex network theory^66,67^.

### Experimental setup for serial response tasks

Subjects performed a self-paced serial reaction time task using a computer screen and keyboard. Each stimulus was presented as a horizontal row of five gray squares; all five squares were shown at all times. The squares corresponded spatially with the keys ‘Space’, ‘H’, ‘J’, ‘K’, and ‘L’, with the left square representing ‘Space’ and the right square representing ‘L’ (Fig. 1b). To indicate a target key or pair of keys for the subject to press, the corresponding squares would become outlined in red (Fig. 1a). When subjects pressed the correct key combination, the squares on the screen would immediately display the next target. If an incorrect key or pair of keys was pressed, the message ‘Error!’ was displayed on the screen below the stimuli and remained until the subject pressed the correct key(s). The order in which stimuli were presented to each subject was prescribed by either a random walk or a Hamiltonian walk on a graph of *N* = 15 nodes, and each sequence consisted of 1500 stimuli. For each subject, one of the 15 key combinations was randomly assigned to each node in the graph (Fig. 1a). Across all graphs, each node was connected to its four neighboring nodes with a uniform 0.25 transition probability. Importantly, given the uniform edge weights and homogeneous node degrees (*k* = 4), the only differences between the transition graphs lay in their higher-order structure.

In the first experiment, we presented subjects with random walk sequences drawn from two different graph topologies: a modular graph with three communities of five densely connected nodes and a lattice graph representing a 3 × 5 grid with periodic boundary conditions (Fig. 1c). The purpose of this experiment was to demonstrate the systematic dependencies of human reaction times on higher-order network structure, following similar results reported in recent literature^22,37^. In particular, we demonstrate two higher-order network effects: In the cross-cluster surprisal effect, average reaction times for within-cluster transitions in the modular graph are significantly faster than reaction times for between-cluster transitions (Fig. 2a); and in the modular-lattice effect, average reaction times in the modular graph are significantly faster than reaction times in the lattice graph (Fig. 2b).

In the second experiment, we presented subjects with Hamiltonian walk sequences drawn from the modular graph. Specifically, each sequence consisted of 700 random walk trials (intended to allow each subject to learn the graph structure), followed by eight repeats of 85 random walk trials and 15 Hamiltonian walk trials (Supplementary Discussion)^20^. Importantly, we find that the cross-cluster surprisal effect remains significant within the Hamiltonian walk trials (Fig. 2a).

In the third experiment, we considered a ring graph where each node was connected to its nearest and next-nearest neighbors in the ring (Fig. 6a). In order to study the dependence of human expectations on violations to the network structure, the first 500 trials for each subject constituted a standard random walk, allowing each subject time to develop expectations about the underlying transition structure. Across the final 1000 trials, we randomly distributed 50 network violations: 20 short violations of topological distance two and 30 long violations, 20 of topological distance three and 10 of topological distance four (Fig. 6a). As predicted by our model, we found a novel violations effect, wherein violations of longer topological distance give rise to larger increases in reaction times than short, local violations (Fig. 6b, c).

### Data analysis for serial response tasks

To make inferences about subjects’ internal expectations based on their reaction times, we used more stringent filtering techniques than previous experiments when pre-processing the data^22^. Across all experiments, we first excluded from analysis the first 500 trials, in which subjects’ reaction times varied wildly (Fig. 1e), focusing instead on the final 1000 trials (or simply on the Hamiltonian trials in the second experiment), at which point subjects had already developed internal expectations about the transition structures. We then excluded all trials in which subjects responded incorrectly. Finally, we excluded reaction times that were implausible, either three standard deviations from a subject’s mean reaction time or below 100 ms. Furthermore, when measuring the network effects in all three experiments (Figs. 3 and 6), we also excluded reaction times over 3500 ms for implausibility. When estimating the parameters of our model and measuring model performance in the first two experiments (Fig. 4), to avoid large fluctuations in the results based on outlier reactions, we were even more stringent, excluding all reaction times over 2000 ms. Taken together, when measuring the cross-cluster surprisal and modular-lattice effects (Fig. 2), we used an average of 931 trials per subject; when estimating and evaluating our model (Fig. 4), we used an average of 911 trials per subject; and when measuring the violation effects (Fig. 6), we used an average of 917 trials per subject. To ensure that our results are robust to particular choices in the data processing, we additionally studied all 1500 trials for each subject rather than just the final 1000, confirming that both the cross-cluster surprisal and modular-lattice effects remain significant across all trials (Supplementary Tables 6 and 7).

### Measurement of network effects using mixed effects models

In order to extract the effects of higher-order network structure on subjects’ reaction times, we used linear mixed effects models, which have become prominent in human research where many measurements are made for each subject^38,68^. Put simply, mixed effects models generalize standard linear regression techniques to include both fixed effects, which are constant across subjects, and random effects, which vary between subjects. Compared with standard linear models, mixed effects models allow for differentiation between effects that are subject-specific and those that persist across an entire population. Here, all models were fit using the fitlme function in MATLAB (R2018a), and random effects were chosen as the maximal structure that (i) allowed model convergence and (ii) did not include effects whose 95% confidence intervals overlapped with zero^69^. In what follows, when defining mixed effects models, we employ the standard R notation^70^.

First, we considered the cross-cluster surprisal effect (Fig. 2a). As we were only interested in measuring higher-order effects of the network topology on human reaction times, it was important to regress out simple biomechanical dependencies on the target button combinations (Fig. 1d), the natural quickening of reactions with time (Fig. 1e), and the effects of recency on reaction times^39,40^. Also, for the first experiment, since some subjects responded to both the modular and lattice graphs (Experimental Procedures), it was important to account for changes in reaction times due to which stage of the experiment a subject was in. To measure the cross-cluster surprisal effect, we fit a mixed effects model with the formula ‘RT~log(Trial)*Stage+Target+Recency+Trans_Type+(1+log(Trial)*Stage+Recency+Trans_Type|ID)’, where RT is the reaction time, Trial is the trial number (we found that log(Trial) was far more predictive of subjects’ reaction times than the trial number itself), Stage is the stage of the experiment (either one or two), Target is the target button combination, Recency is the number of trials since the last instance of the current stimulus, Trans_Type is the type of transition (either within-cluster or between-cluster), and ID is each subject’s unique ID. Fitting this mixed effects model to the random walk data in the first experiment (Supplementary Table 1), we found a 35 ms increase in reaction times (*p* < 0.001, *F*-test) for between-cluster transitions relative to within-cluster transitions (Fig. 2a). Similarly, fitting the same mixed effects model but without the variable Stage to the Hamiltonian walk data in the second experiment (Supplementary Table 4), we found a 36 ms increase in reaction times (*p* < 0.001, *F*-test) for between- versus within-cluster transitions (Fig. 2a). We note that because reaction times are not Gaussian distributed, it is fairly standard to perform a log transformation. However, for the above result as well as those that follow, we find the same qualitative effects with or without a log transformation.

Second, we studied the modular-lattice effect (Fig. 2b). To do so, we fit a mixed effects model with the formula ‘RT~log(Trial)*Stage+Target+Recency+Graph+(1+log(Trial)*Stage+Recency+Graph|ID)’, where Graph represents the type of transition network, either modular or lattice. Fitting this mixed effects model to the data in the first experiment (Supplementary Table 2), we found a fixed 23 ms increase in reaction times (*p* < 0.001, *F*-test) in the lattice graph relative to the modular graph (Fig. 2b).

Finally, we considered the effects of violations of varying topological distance in the ring lattice (Fig. 6c). We fit a mixed effects model with the formula ‘RT~log(Trial)+Target+Recency+Top_Dist+(1+log(Trial)+Recency+Top_Dist|ID)’, where Top_Dist represents the topological distance of a transition, either one for a standard transition, two for a short violation, or three for a long violation. Fitting the model to the data in the third experiment (Supplementary Tables 10 and 11), we found a 38 ms increase in reaction times for short violations relative to standard transitions (*p* < 0.001, *F*-test), a 63 ms increase in reaction times for long violations relative to standard transitions (*p* < 0.001, *F*-test), and a 28 ms increase in reaction times for long violations relative to short violations (*p* = 0.011, *F*-test). Put simply, people are more surprised by violations to the network structure that take them further from their current position in the network, suggesting that people have an implicit understanding of the topological distances between nodes in the network.

### Estimating parameters and making quantitative predictions

Given an observed sequence of nodes *x*_1_, …, *x*_*t*−1_, and given an inverse temperature *β*, our model predicts the anticipation, or expectation, of the subsequent node *x*_*t*_ to be $a\left( t \right) = \hat A_{x_{t - 1},x_t}\left( {t - 1} \right)$. In order to quantitatively describe the reactions of an individual subject, we must relate the expectations *a*(*t*) to predictions about a person’s reaction times $\hat r\left( t \right)$ and then calculate the model parameters that best fit the reactions of an individual subject. The simplest possible prediction is given by the linear relation $\hat r\left( t \right) = r_0 + r_1a\left( t \right)$, where the intercept *r*_0_ represents a person’s reaction time with zero anticipation and the slope *r*_1_ quantifies the strength with which a person’s reaction times depend on their internal expectations.

In total, our predictions $\hat r\left( t \right)$ contain three parameters (*β*, *r*_0_, and *r*_1_), which must be estimated from the reaction time data for each subject. Before estimating these parameters, however, we first regress out the dependencies of each subject’s reaction times on the button combinations, trial number, and recency using a mixed effects model of the form ‘RT~log(Trial)*Stage+Target+Recency+(1+log(Trial)*Stage+Recency|ID)’, where all variables were defined in the previous section. Then, to estimate the model parameters that best describe an individual’s reactions, we minimize the RMS prediction error with respect to each subject’s observed reaction times, ${\mathrm{RMSE}} = \sqrt {\frac{1}{T}\mathop {\sum}\nolimits_t {\left( {r\left( t \right) - \hat r\left( t \right)} \right)^2} }$, where *T* is the number of trials. We note that, given a choice for the inverse temperature *β*, the linear parameters *r*_0_ and *r*_1_ can be calculated analytically using standard linear regression techniques. Thus, the problem of estimating the model parameters can be restated as a one-dimensional minimization problem; that is, minimizing RMSE with respect to the inverse temperature *β*. To find the global minimum, we began by calculating RMSE along 100 logarithmically spaced values for *β* between 10^−4^ and 10. Then, starting at the minimum value of this search, we performed gradient descent until the gradient fell below an absolute value of 10^−6^. For a derivation of the gradient of the RMSE with respect to the inverse temperature *β*, we point the reader to the Supplementary Discussion. Finally, in addition to the gradient descent procedure described above, for each subject we also manually checked the RMSE associated with the two limits *β* → 0 and *β* → ∞. The resulting model parameters are shown in Fig. 4a, b for random walk sequences and Fig. 4g, h for Hamiltonian walk sequences.

### Experimental setup for *n*-back memory task

Subjects performed a series of *n*-back memory tasks using a computer screen and keyboard. Each subject observed a random sequence of the letters ‘B’, ‘D’, ‘G’, ‘T’, and ‘V’, wherein each letter was randomly displayed in either upper or lower case. The subjects responded on each trial using the keyboard to indicate whether or not the current letter was the same as the letter that occurred *n* trials previously. For each subject, this task was repeated for the conditions *n* = 1, 2, and 3, and each condition consisted of a sequence of 100 letters. The three conditions were presented in a random order to each subject. After the *n*-back task, each subject then performed a serial response task (equivalent to the first experiment described above) consisting of 1500 random walk trials drawn from the modular graph.

### Data analysis for *n*-back memory task

In order to estimate the inverse temperature *β* for each subject from their *n*-back data, we directly measured their memory distribution *P*(Δ*t*). As described in the main text, we treated each positive response indicating that the current stimulus matched the target stimulus as a sample of *P*(Δ*t*) by measuring the distance in trials Δ*t* between the last instance of the current stimulus and the target (Fig. 5a). For each subject, we combined all such samples across the three conditions *n* = 1, 2, and 3 to arrive at a histogram for Δ*t*. In order to generate robust estimates for the inverse temperature *β*, we generated 1000 bootstrap samples of the Δ*t* histogram for each subject. For each sample, we calculated a linear fit to the distribution *P*(Δ*t*) on log-linear axes within the domain 0  ≤  Δ*t*  ≤  4 (note that we could not carry the fit out to Δ*t* = 10 because the data is much sparser for individual subjects). To ensure that the logarithm of *P*(Δ*t*) was well defined for each sample—that is, to ensure that *P*(Δ*t*) > 0 for all Δ*t*—we added one count to each value of Δ*t*. We then estimated the inverse temperature *β* for each sample by calculating the negative slope of the linear fit of log*P*(Δ*t*) versus Δ*t*. To arrive at an average estimate of *β* for each subject, we averaged *β* across the 1000 bootstrap samples. Finally, we compared these estimates of *β* from the *n*-back experiment with estimates of *β* from subjects’ reaction times in the subsequent serial response task, as described above. We found that these two independent estimates of people’s inverse temperatures are significantly correlated (excluding subjects for which *β* = 0 or *β* → ∞), with a Spearman coefficient *r*_s_ = 0.28 (*p* = 0.047, permutation test). We note that we do not use the Pearson correlation coefficient because the estimates for *β* are not normally distributed for either the reaction time task (*p* < 0.001) nor the *n*-back task (*p* = 0.013) according to the Anderson–Darling test^58^. This non-normality can be clearly seen in the distributions of *β* in Fig. 4a, g.

### Experimental procedures

All participants provided informed consent in writing and experimental methods were approved by the Institutional Review Board of the University of Pennsylvania. In total, we recruited 634 unique participants to complete our studies on Amazon’s Mechanical Turk. For the first serial response experiment, 101 participants only responded to sequences drawn from the modular graph, 113 participants only responded to sequences drawn from the lattice graph, and 72 participants responded to sequences drawn from both the modular and lattice graphs in back-to-back (counter-balanced) sessions for a total of 173 exposures to the modular graph and 185 exposures to the lattice graph. For the second experiment, we recruited 120 subjects to respond to random walk sequences with Hamiltonian walks interspersed, as described in the Supplementary Discussion. For the third experiment, we recruited 78 participants to respond to sequences drawn from the ring graph with violations randomly interspersed. For the *n*-back experiment, 150 subjects performed the *n*-back task and, of those, 88 completed the subsequent serial response task. Worker IDs were used to exclude duplicate participants between experiments, and all participants were financially remunerated for their time. In the first experiment, subjects were paid up to $11 for up to an estimated 60 min: $3 per network for up to two networks, $2 per network for correctly responding on at least 90% of the trials, and $1 for completing the entire task. In the second and third experiments, subjects were paid up to $7.50 for an estimated 30 min: $5.50 for completing the experiment and $2 for correctly responding on at least 90% of the trials. In the *n*-back experiment, subjects were paid up to $8.50 for an estimated 45 min: $7 for completing the entire experiment and $1.50 for correctly responding on at least 90% of the serial response trials.

At the beginning of each experiment, subjects were provided with the following instructions: “In a few minutes, you will see five squares shown on the screen, which will light up as the experiment progresses. These squares correspond with keys on your keyboard, and your job is to watch the squares and press the corresponding key when that square lights up”. For the 72 subjects that responded to both the modular and lattice graphs in the first experiment, an additional piece of information was also provided: “This part will take around 30 minutes, followed by a similar task which will take another 30 minutes”. Before each experiment began, subjects were given a short quiz to verify that they had read and understood the instructions. If any questions were answered incorrectly, subjects were shown the instructions again and asked to repeat the quiz until they answered all questions correctly. Next, all subjects were shown a 10-trial segment that did not count towards their performance; this segment also displayed text on the screen explicitly telling the subject which keys to press on their keyboard. Subjects then began their 1500-trial experiment. For the subjects that responded to both the modular and lattice graphs, a brief reminder was presented before the second graph, but no new instructions were given. After completing each experiment, subjects were presented with performance information and their bonus earned, as well as the option to provide feedback.

### Reporting summary

Further information on research design is available in the Nature Research Reporting Summary linked to this article.

## Supplementary information

Supplementary Information

Reporting Summary

## Data Availability

Source data for Fig. 1 are provided in Supplementary Data File 1. Source data for Fig. 2, Supplementary Figs. 2 and 3, and Supplementary Tables 1–9 are provided in Supplementary Data File 2. Source data for Fig. 5 are provided in Supplementary Data File 3. Source data for Fig. 6, Supplementary Figs. 4 and 5, and Supplementary Tables 10 and 11 are provided in Supplementary Data File 4. Source data from Supplementary Fig. 1 are provided in Supplementary Data File 5.

## Source data

Source Data

## Code availability

The code that supports the findings of this study is available from the corresponding author upon reasonable request.
