# Supplementary material for: Abstract representations of events arise from mental errors in learning and memory
Source: Nat Commun. 2020 May 8;11:2313. doi: 10.1038/s41467-020-15146-7 (PMC7210268; doi:10.1038/s41467-020-15146-7)
Supplement: Supplementary file 1 — Supplementary Information [file 41467_2020_15146_MOESM1_ESM.pdf]

Abstract representations of events arise from mental errors in learning and memory

Lynn et al.

Supplementary Information

Structure from noise: Mental errors yield abstract representations of events

Christopher W. Lynn[1], Ari E. Kahn[2,3], Nathaniel Nyema[3], & Danielle S. Bassett[1,3,4,5,6,7,*]

[1]*Department of Physics & Astronomy, College of Arts & Sciences, University of Pennsylvania, Philadelphia, PA 19104, USA*

[2]*Department of Neuroscience, Perelman School of Medicine, University of Pennsylvania, Philadelphia, PA 19104, USA*

[3]*Department of Bioengineering, School of Engineering & Applied Science, University of Pennsylvania, Philadelphia, PA 19104, USA*

[4]*Department of Electrical & Systems Engineering, School of Engineering & Applied Science, University of Pennsylvania, Philadelphia, PA 19104, USA*

[5]*Department of Neurology, Perelman School of Medicine, University of Pennsylvania, Philadelphia, PA 19104, USA*

[6]*Department of Psychiatry, Perelman School of Medicine, University of Pennsylvania, Philadelphia, PA 19104, USA*

[7]*Santa Fe Institute, Santa Fe, NM 87501, USA*

Contents

1 Introduction **4**

2 The effects of node heterogeneity on human expectations **5**

3 Measuring higher-order network effects **7**

3.1 Cross-cluster surprisal effect 8

3.2 Modular-lattice effect 8

4 Cross-cluster surprisal with Hamiltonian walks **9**

4.1 Removing Hamiltonian trials before the first cross-cluster transition 12

4.2 Decreasing cross-cluster surprisal with increasing Hamiltonian trials 13

4.3 Experimental setup and procedures 14

5 Controlling for recency in random walks **15**

5.1 Cross-cluster surprisal effect while controlling for recency 15

5.2 Modular-lattice effect while controlling for recency 17

6 Measuring network effects including early trials **19**

6.1 Cross-cluster surprisal effect with early trials 19

6.2 Modular-lattice effect with early trials 19

7 Network effects on error trials **21**

7.1 Cross-cluster surprisal effect on errors 22

7.2 Modular-lattice effect on errors 22

8 Measuring the effects of network violations **23**

9 Controlling for recency: Network violations **25**

10 The forgetting of stimuli cannot explain network effects **28**

11 Gradient of RMS error with respect to inverse temperature β **30**

12 Connection to the successor representation **31**

References **34**

1 Introduction

In this Supplementary Information, we provide extended discussion and data to support the results presented in the main text. The content is organized to roughly mirror the organization of the paper. In Sec. 2, we present experimental evidence that human reaction times – in addition to depending on higher-order network features – also reflect differences in fine-scale structure at the level of individual nodes. Just as for the higher-order effects presented in the main text, we demonstrate that these fine-scale phenomena are accurately predicted by our maximum entropy model. In Sec. 3, we present the mixed effects models that were used to estimate the cross-cluster surprisal and modular-lattice effects. In Sec. 5, we demonstrate that the cross-cluster surprisal and modular-lattice effects cannot simply be explained by recency by directly controlling for the recency of stimuli. In Sec. 4, we use Hamiltonian walks to experimentally control for recency. In Sec. 6, we show that the cross-cluster surprisal and modular-lattice effects persist even when considering all 1500 trials for each subject. In Sec. 7, we show that the probability of an error on the serial response tasks increases for between- versus within-cluster transitions in the modular graph, indicating that the free energy framework can be used to predict human behaviors beyond reaction times. In Sec. 8, we present the mixed effects models that were used to estimate the effects of violations in the ring graph. In Sec. 9, we show that the effects of network violations cannot be explained by recency alone. In Sec. 10, we discuss why the forgetting of past stimuli altogether cannot explain the higher-order network effects that we examine in the main text. In Sec. 11, to aid in the reconstruction of our gradient descent algorithm for estimating the inverse temperature β from subjects' reaction times, we derive an analytic form for the gradient of the RMS prediction

error of our model with respect to β. In Sec. 12, we discuss the relationship between our model and the successor representation in reinforcement learning.

2 The effects of node heterogeneity on human expectations

In the main text, we demonstrated that human expectations depend critically on the higher-order network structure of transitions. In addition to these higher-order phenomena, it has long been known that human expectations also reflect differences in the fine-scale structure of transition networks.[1,2] For instance, humans are surprised by rare transitions, represented in a transition network by edges with low probability weight.[3] Here, we provide empirical evidence showing that people's expectations also depend on the local topologies of the nodes that bookend a transition, and that these fine-scale effects are consistently predicted by our maximum entropy model.

In order to clearly study the effects of higher-order network structure, in the main text we focused on networks with uniform edge weights and node degrees. Here, to study the effects of node heterogeneity, we instead consider a set of Erdös-Rényi random graphs with the same number of nodes ($N = 15$) and edges (30) as in our previous modular and lattice graphs. To ensure that the random walks are properly defined, we set the transition probability A_{ij} of each edge in the graph to $1/k_i$, where k_i is the degree of node i. Since the probabilities A_{ij} decrease as the degree k_i increases, one should suspect that high-degree (or hub) nodes yield decreased anticipations – and therefore increased reaction times – at the next step of a random sequence. Indeed, using Eq. (6) from the main text, we find that our model analytically predicts decreased expectations following a high-degree node (Supplementary Fig. 1a). Furthermore, across 177 human subjects, we find a

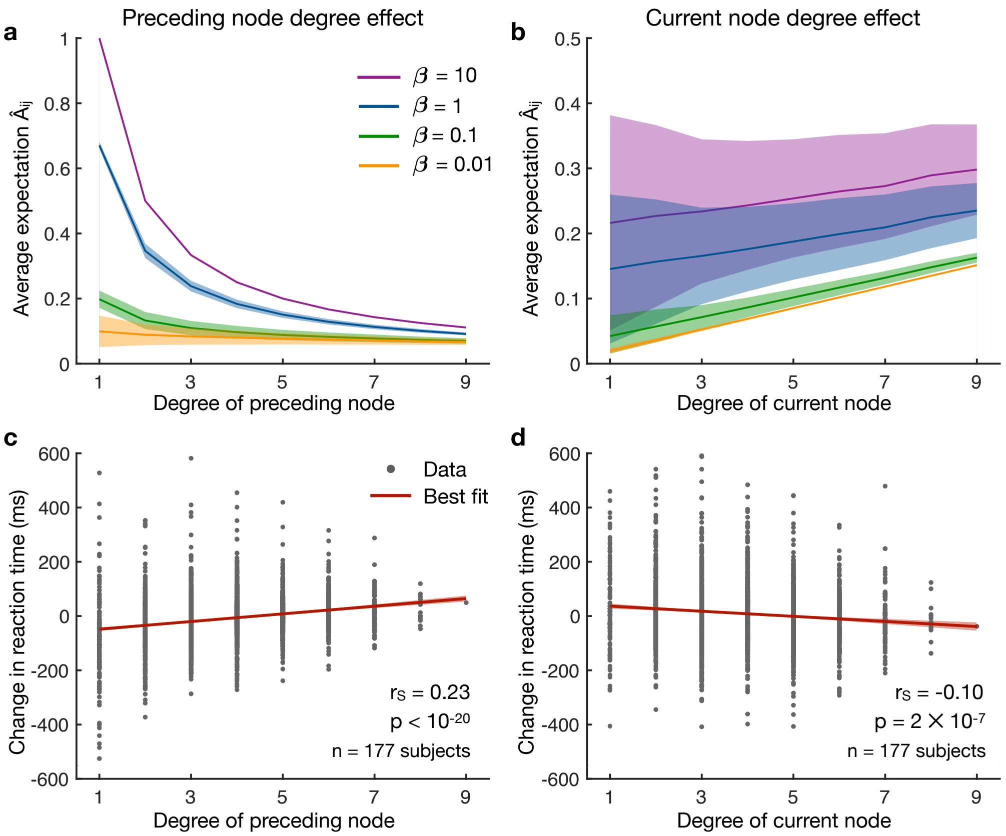

Supplementary Fig. 1: The effects of node degree on reaction times.

a, The average expectation $\hat{A}_{ij}$ plotted with respect to the degree of the preceding node i across a range of inverse temperatures β. As expected, expectations decrease as the degree of the preceding node increases; and for $\beta = 10$, we have $\hat{A}_{ij} \approx A_{ij} = 1/k_i$. The lines and shaded regions represent averages and 95% confidence intervals over 1000 randomly-generated Erdös-Rényi networks. **b**, People exhibit sharp increases in reaction time following nodes of higher degree, with Spearman's correlation $r_S = 0.23$. The data is combined across 177 subjects, each of whom was asked to respond to a sequence of 1500 stimuli drawn from a random Erdös-Rényi network. Each data point represents the average reaction time for one node of a graph, and so each subject contributes 15 points. The line and shaded region represent the best fit and 95% confidence interval, respectively. **c**, The average expectation $\hat{A}_{ij}$ plotted with respect to the degree of the current node j across the same range of inverse temperatures as in **a**. **d**, People exhibit a steady decline in reaction times as the current node degree increases, with Spearman's correlation $r_S = -0.10$. Source data are provided as a Source Data file.

strong positive correlation between people's reaction times and the degree of the preceding node in the sequence (Supplementary Fig. 1b).

Interestingly, while people's anticipations exhibit a sharp decline if the preceding node has high-degree, our model predicts that these hub nodes instead yield increased anticipations on the current step (Supplementary Fig. 1c). Thus, while hub nodes give rise to marked increases in reaction times on the subsequent step, these high-degree nodes actually yield faster reactions on the current step[2] (Supplementary Fig. 1d). This juxtaposition of effects from one time step to the next highlights the complex ways in which the network structure of transitions can affect people's mental representations. Additionally, the success of our model in predicting these competing phenomena further strengthens our conclusion that mental errors play a crucial role in shaping people's internal expectations.

3 Measuring higher-order network effects

In order to extract the effects of higher-order network structure on subjects' reaction times, we use linear mixed effects models, which have become prominent in human research where many measurements are made for each subject.[4,5] To fit our mixed effects models and to estimate the statistical significance of each effect we use the `fitlme` function in MATLAB (R2018a). In what follows, when referring to our mixed effects models, we adopt the standard R notation.[6]

3.1 Cross-cluster surprisal effect

We first measure the cross-cluster surprisal effect (Fig. 2a) using a mixed effects model with the formula 'RT $\sim \log(\text{Trial}) * \text{Stage} + \text{Target} + \text{Recency} + \text{Trans\_Type} + (1 + \log(\text{Trial}) * \text{Stage} + \text{Recency} + \text{Trans\_Type} \,|\, \text{ID})$', where RT is the reaction time, Trial is the trial number between 501 and 1500, Stage is the stage of the experiment (either one or two), Target is the target button combination, Recency is the number of trials since last observing a node,[7] Trans_Type is the type of transition (either within-cluster or between-cluster), and ID is each subject's unique ID. We remark that our inclusion of Recency in the model is intended to distinguish the graph effects that we are interested in studying from the possible confound of recency, an effect that we directly control for in Sec. 5. The mixed effects model is summarized in Supplementary Table 1, reporting a 35 ms increase in reaction times for between-cluster transitions relative to within-cluster transitions (Fig. 2a). This result is measured from the reaction time data for all 173 subjects that observed random walks in the modular graph.

3.2 Modular-lattice effect

We next measure the modular-lattice effect (Fig. 2b) using a mixed effects model of the form 'RT $\sim \log(\text{Trial}) * \text{Stage} + \text{Target} + \text{Recency} + \text{Graph} + (1 + \log(\text{Trial}) * \text{Stage} + \text{Recency} \,|\, \text{ID})$', where Graph represents the type of transition network, either modular or lattice. Note that we only include Graph as a fixed effect because the corresponding mixed effect is not statistically significant. The mixed effects model is summarized in Supplementary Table 2, reporting a 23 ms increase in reaction times in the lattice graph relative to the modular graph (Fig. 2b). This result is

Effect	Estimate (ms)	t-value	Pr(>|t|)	Significance
(Intercept)	1418.9 ± 73.1	19.42	< 0.001	$***$
$\log$(Trial)	-92.1 ± 9.2	-9.96	< 0.001	$***$
Stage	-551.5 ± 85.0	-6.48	< 0.001	$***$
Recency	1.4 ± 0.1	23.57	< 0.001	$***$
Trans_Type	34.9 ± 6.0	5.77	< 0.001	$***$
$\log$(Trial):Stage	67.0 ± 11.4	5.89	< 0.001	$***$

Supplementary Table 1: Mixed effects model measuring the cross-cluster surprisal effect. A mixed effects model fit to the reaction time data for the modular graph with the goal of measuring the cross-cluster surprisal effect. We find a significant 35 ms increase in reaction times (173 subjects) for between-cluster transitions versus within-cluster transitions (grey). The significance column represents p-values less than 0.001 ($***$), less than 0.01 ($**$), and less than 0.05 ($*$). Source data are provided as a Source Data file.

measured from the reaction time data for the 72 subjects that observed random walks in both the modular and lattice graphs.

4 Cross-cluster surprisal with Hamiltonian walks

Throughout the main text, we assume that people's reaction times reflect their internal representations of the transition structure. To justify this assumption, we must show that the higher-order network effects cannot simply be explained by recency. Here, we measure the cross-cluster surprisal effect while experimentally controlling for recency using Hamiltonian walks. In contrast to random walks, Hamiltonian walks visit each node in the transition graph exactly once, thereby

Effect	Estimate (ms)	t-value	Pr(>|t|)	Significance
(Intercept)	1436.5 ± 48.4	29.67	< 0.001	$***$
$\log$(Trial)	-97.2 ± 6.1	-15.89	< 0.001	$***$
Stage	-555.2 ± 59.3	-9.36	< 0.001	$***$
Recency	1.7 ± 0.1	29.94	< 0.001	$***$
Graph	22.8 ± 5.8	3.95	< 0.001	$***$
$\log$(Trial):Stage	71.4 ± 8.4	8.48	< 0.001	$***$

Supplementary Table 2: Mixed effects model measuring the modular-lattice effect. A mixed effects model fit to the reaction time data for the modular and lattice graphs with the goal of measuring the modular-lattice effect. We find a significant 23 ms increase in reaction times overall (72 subjects) in the lattice graph relative to the modular graph (grey). The significance column represents p-values less than 0.001 ($***$), less than 0.01 ($**$), and less than 0.05 ($*$). Source data are provided as a Source Data file.

guaranteeing that each node is visited once every 15 trials. We run a new experiment in which each subject (out of 120 subjects) is presented with a sequence of 1500 stimuli drawn from the modular graph: The first 700 nodes reflect a standard random walk, while the remaining 800 trials consist of 8 repeated segments of 85 stimuli specified by a random walk followed by 15 stimuli specified by a Hamiltonian walk. The initial 700 random walk trials are meant to constitute a learning phase in which the subject builds an internal representation of the modular graph. Since, in the modular graph, Hamiltonian walks do not obey the same transition probabilities as random walks, the sequences of 85 random walk trials between each Hamiltonian sequence are meant to help the subject maintain their learned representation. Within the set of Hamiltonian walks through the

Effect	Estimate (ms)	t-value	Pr(>|t|)	Significance
(Intercept)	1420.8 ± 162.3	8.75	< 0.001	$* * *$
$\log$(Trial)	-101.4 ± 22.7	-4.48	< 0.001	$* * *$
Recency	0.6 ± 0.1	5.00	< 0.001	$* * *$
Trans_Type	35.6 ± 13.7	2.59	0.010	$**$

Supplementary Table 3: Mixed effects model measuring the cross-cluster surprisal effect in Hamiltonian walks. A mixed effects model fit to subjects' reaction times in Hamiltonian walks on the modular graph with the goal of measuring the cross-cluster surprisal effect. We find a significant 36 ms increase in reaction times (120 subjects) for between-cluster transitions versus within-cluster transitions (grey). The significance column represents p-values less than 0.001 ($* * *$), less than 0.01 ($**$), and less than 0.05 ($*$). Source data are provided as a Source Data file.

modular graph, the probability of transitioning from one cluster boundary node to the adjacent one (if not already visited) is 1, whereas the probability of transitioning from the latter boundary node to each of the adjacent non-boundary nodes is 1/3. To eliminate this difference, we randomly selected one fixed Hamiltonian walk for each subject. This fixed walk was entered at a different node depending on where the preceding walk terminated, and we randomly switched between forward and backward traversals for each walk.[8]

We measure the cross-cluster surprisal within the Hamiltonian trials using a mixed effects model with the formula 'RT $\sim$ $\log$(Trial) + Target + Recency + Trans_Type + $(1 + \log$(Trial) + Recency + Trans_Type $|$ ID$)$', where each of the variables has been defined previously. The model is summarized in Supplementary Table 3, reporting a 36 ms increase in reaction times for between-

cluster transitions relative to within-cluster transitions within Hamiltonian trials ($p = 0.010$), matching (within errors) the effect size reported in the original experiment that only included random walks (see Supplementary Table 1). This result is measured from the reaction time data for all 120 subjects that observed random walks with Hamiltonian walks interspersed in the modular graph. This result indicates that the cross-cluster surprisal effect cannot be explained by recency alone, and must therefore must be at least partially driven by people's internal representations of the transition structure.

4.1 Removing Hamiltonian trials before the first cross-cluster transition

The purpose of the Hamiltonian walk experiment described above is to experimentally control for the effects of recency on people's reaction times. However, when thinking carefully about the transition from a random walk to a Hamiltonian walk, it becomes clear that recency might still have a noticeable impact. Consider, for example the last few trials of a random walk preceding a transition to a Hamiltonian walk – the corresponding stimuli are likely to belong to the same module in the modular graph. When the sequence converts to a Hamiltonian walk, the first few stimuli are also likely to belong to the same module, thereby inducing a decrease in reaction times due to recency. Therefore, in order to more thoroughly control for recency effects, we considered only trials after the first cross-cluster transition within each Hamiltonian walk. We carry out this restricted analysis using the same form for the mixed effects model as that described above: 'RT $\sim$ $\log$(Trial) + Target + Recency + Trans_Type + $(1 + \log$(Trial) + Recency + Trans_Type | ID)'. The model, which is summarized in Supplementary Table 4, estimates a significant cross-cluster surprisal effect of 28 ms ($p = 0.043$), again matching within errors the effect size found in the

Effect	Estimate (ms)	t-value	Pr(>|t|)	Significance
(Intercept)	1536.6 ± 178.0	8.63	< 0.001	$***$
$\log$(Trial)	-116.3 ± 24.8	-4.68	< 0.001	$***$
Recency	0.4 ± 0.1	3.00	0.003	$**$
Trans_Type	28.2 ± 13.9	2.03	0.043	$*$

Supplementary Table 4: Mixed effects model measuring the cross-cluster surprisal effect in restricted Hamiltonian walks. A mixed effects model fit to subjects' reaction times after the first cross-cluster transition within each Hamiltonian walk. We find a significant 28 ms increase in reaction times (120 subjects) for between-cluster transitions versus within-cluster transitions (grey). The significance column represents p-values less than 0.001 ($***$), less than 0.01 ($**$), and less than 0.05 ($*$). Source data are provided as a Source Data file.

original random walk data.

4.2 Decreasing cross-cluster surprisal with increasing Hamiltonian trials

As discussed above, the first 700 trials of each sequence were drawn from a random walk to allow subjects to build an internal representation of the random walk transition structure. Since the transition probabilities reflected in the Hamiltonian walks differ from those in the random walks, we expect subjects' representations of the transition structure to shift as they observe increasing numbers of Hamiltonian trials. Therefore, to further establish the notion that people's reactions are primarily driven by their internal representations, here we show that the strength of the cross-cluster surprisal decreases as subjects observe increasing numbers of Hamiltonian trials. To do so, we use a mixed effects model with the formula 'RT $\sim \log(\text{Trial}) * \text{Trans\_Type} + \text{Target} + \text{Recency} +$

Effect	Estimate (ms)	t-value	Pr(>|t|)	Significance
(Intercept)	1394.1 ± 188.8	7.39	< 0.001	$***$
$\log$(Trial)	-96.1 ± 26.4	-3.64	< 0.001	$***$
Recency	0.4 ± 0.1	3.02	0.003	$**$
Trans_Type	640.3 ± 271.9	2.35	0.019	$*$
$\log$(Trial):Trans_Type	-87.2 ± 38.7	-2.25	0.024	$*$

Supplementary Table 5: Mixed effects model measuring the decrease in cross-cluster surprisal with increasing Hamiltonian trials. A mixed effects model fit to subjects' reaction times in Hamiltonian walks on the modular graph with the goal of measuring the dependence of the cross-cluster surprisal on increasing trial number. We find a significant decrease in the strength of the cross-cluster surprisal with increasing trials (grey), indicating that the introduction of Hamiltonian walks weakens people's internal representations of the random walk structure (120 subjects). The significance column represents p-values less than 0.001 ($***$), less than 0.01 ($**$), and less than 0.05 ($*$). Source data are provided as a Source Data file.

$(1 + \log(\text{Trial}) + \text{Recency} + \text{Trans\_Type} \,|\, \text{ID})$', where the only difference with the formula above is that here we include an interaction term between $\log(\text{Trial})$ and Trans_Type. The results of the fitted model are summarized in Supplementary Table 5, reporting a significant decrease in the strength of the cross-cluster surprisal with increasing Hamiltonian trials ($p = 0.024$).

4.3 Experimental setup and procedures

Subjects performed a self-paced serial reaction time task, as described in the Methods section of the main text. The only difference between this experiment and the original random walk experiments is that the 1500 trials were split into 700 trials drawn as a random walk and a subsequent 800 trials

divided into 8 segments of 85 random walk trials followed by 15 Hamiltonian walk trials, all drawn from the modular graph. In total, we recruited 120 subjects to perform this Hamiltonian walk experiment, and they were paid up to \$5 each for an estimated 30 minutes: \$3.50 for completing the task and \$1.50 for correctly responding on at least 90% of the trials.

5 Controlling for recency in random walks

In the previous section, we showed that cross-cluster surprisal remains significant during Hamiltonian walks, which experimentally control for the recency of stimuli. Building upon this result, in this section we measure the cross-cluster surprisal and modular-lattice effects in our initial random walk data while filtering our data based on stimulus recency.

5.1 Cross-cluster surprisal effect while controlling for recency

In order to control for recency, we filter our data to only include trials in which the current stimulus was last seen a specific number of trials in the past. For example, when studying trials with a recency of four, we only consider reaction times from our experiments for which the current stimulus was last seen four trials previously. After filtering the data, we then estimate the cross-cluster surprisal effect using a mixed effects model of the form 'RT $\sim \log(\text{Trial}) * \text{Stage} + \text{Target} + \text{Trans\_Type} + (1 + \log(\text{Trial}) * \text{Stage} + \text{Trans\_Type} \mid \text{ID})$'. Supplementary Fig. 2a shows the estimated increase in reaction times for within-cluster versus between-cluster transitions after controlling for recency. Specifically, we consider recency values of two (the minimum) through nine, and we also consider trials with recency greater than or equal to 10, for which the effects of recency should be small. We remark that we do not include trials of recency three in our analysis because, due to the

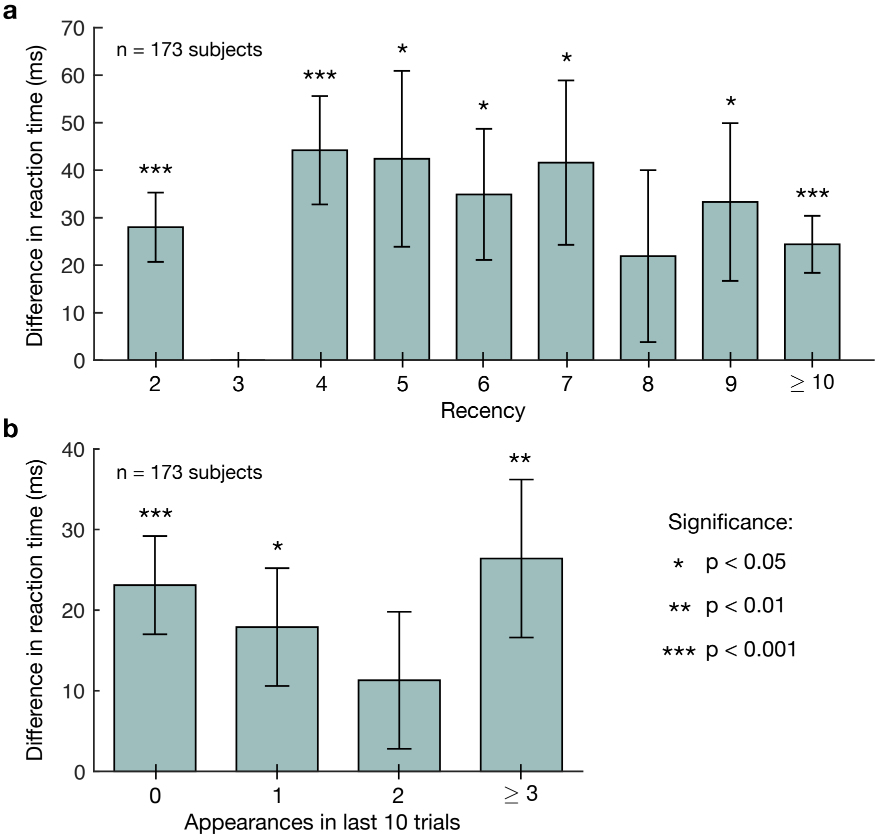

Supplementary Fig. 2: Cross-cluster surprisal while controlling for recency.

a, Increase in reaction times for between-cluster versus within-cluster transitions in the modular graph after controlling for the recency of stimuli. We note that, due to the topology of the modular graph, there do not exist between-cluster transitions with recency three. We find significant cross-cluster surprisal effects for all recency values besides eight. **b**, Increase in reaction times for between- versus within-cluster transitions after controlling for the number of times that the current stimulus has appeared in the previous 10 trials. We observe significant cross-cluster surprisal for all numbers of recent stimulus appearances besides two. Effect sizes (represented by bar plots), standard deviations (represented by error bars), and *p*-values are estimated using mixed effects models. The results are measured for all 173 subjects that observed random walks in the modular graph. Source data are provided as a Source Data file.

topology of the modular graph, there do not exist between-cluster transitions with recency three. We find significant effects for all recency values besides eight.

In addition to controlling for the recency of stimuli, we also study the cross-cluster surprisal while controlling for the number of appearances of the current stimulus in the last 10 trials. In particular, we filter our data to only include trials for which the current stimulus was seen a specified number of times in the previous 10 trials, and for each set of filtered data we estimate the cross-cluster surprisal using a mixed effects model of the same form as above. We observe a significant increase in reaction times for between- versus within-cluster transitions for all trials except for those for which the stimulus appeared twice in the last 10 trials (Supplementary Fig. 2b). Together, these results demonstrate that the cross-cluster surprisal effect cannot be explained by recency alone, and therefore must stem, at least in part, from people's internal representations of the transition structure.

5.2 Modular-lattice effect while controlling for recency

We next consider the modular-lattice effect after controlling for recency. Filtering the data from the modular and lattice graphs to only include trials of a given recency, we estimate the difference in reaction times between the two graphs using a mixed effects model of the form '$\text{RT} \sim \log(\text{Trial}) * \text{Stage} + \text{Target} + \text{Graph} + (1 + \log(\text{Trial}) * \text{Stage} \,|\, \text{ID})$'. Supplementary Fig. 3a shows that we find a significant increase in reaction times for the lattice graph relative to the modular graph for all recency values considered besides three, nine, and ≥ 10. Additionally, in Supplementary Fig. 3b, we control for the number of appearances of the current stimulus in the previous 10 trials. Using a mixed effects model of the same form as that above, we find a significant modular-lattice effect in two of the four conditions. Together, these results demonstrate that the difference in reaction times between the modular and lattice graphs persists after controlling for the recency of stimuli,

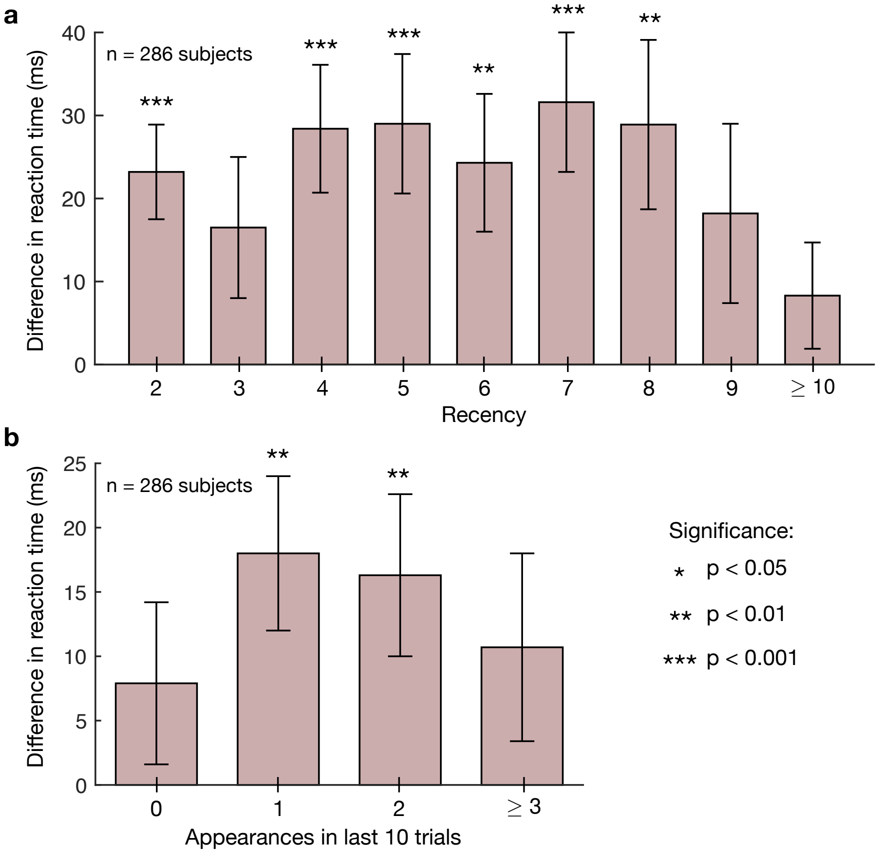

Supplementary Fig. 3: Modular-lattice effect while controlling for recency.

a, Difference in reaction times between the lattice and modular graphs after controlling for the recency of stimuli. We observe a significant increase in reaction times for the lattice graph relative to the modular graph for all recency values besides three, nine, and ≥ 10. **b**, Difference in reaction times between the lattice and modular graphs after controlling for the number of times the current stimulus has appeared in the previous 10 trials. We find a significant modular-lattice effect for one and two stimulus appearances in the last 10 trials. Effect sizes (represented by bar plots), standard deviations (represented by error bars), and *p*-values are estimated using mixed effects models. The results are measured for all 72 subjects that observed random walks in both the modular and lattice graphs. Source data are provided as a Source Data file.

indicating that people are better able to anticipate transitions in the modular graph than in the lattice graph.

6 Measuring network effects including early trials

Throughout the above analysis of the serial response tasks, we purposefully omitted the first 500 trials for each subject, choosing instead to focus on the final 1000 trials. We did this in order to allow the subjects to build an internal representation of each network structure before probing their anticipations of transitions. Here, we show that this data processing step is not necessary to observe higher-order network effects; that is, we show that there exist significant network effects even if we include the first 500 trials in our analysis.

6.1 Cross-cluster surprisal effect with early trials

We first consider the cross-cluster surprisal effect defined by an increase in reaction times for transitions between clusters relative to transitions within clusters in the modular graph. Using a mixed effects model of the same form as that used in the previous analysis in Sec. 3 (i.e., 'RT $\sim \log(\text{Trial}) * \text{Stage} + \text{Target} + \text{Recency} + \text{Trans\_Type} + (1 + \log(\text{Trial}) * \text{Stage} + \text{Recency} + \text{Trans\_Type} \,|\, \text{ID})$'), and including all 1500 trials for each subject, we find a significant 35 ms increase in reaction times for between- versus within-cluster transitions (Supplementary Table 6). We note that this effect is even larger than that observed in our previous analysis (Supplementary Table 1).

6.2 Modular-lattice effect with early trials

We next consider the modular-lattice effect defined by an increase in reaction times in the lattice graph relative to the modular graph. Using a mixed effects model of the same form as that used

Effect	Estimate (ms)	t-value	Pr(>|t|)	Significance
(Intercept)	1340.0 ± 44.4	30.19	< 0.001	$***$
$\log$(Trial)	-88.7 ± 5.0	-17.04	< 0.001	$***$
Stage	-473.1 ± 47.6	-9.93	< 0.001	$***$
Recency	1.5 ± 0.1	24.65	< 0.001	$***$
Trans_Type	35.4 ± 6.0	5.94	< 0.001	$***$
$\log$(Trial):Stage	60.4 ± 5.5	11.06	< 0.001	$***$

Supplementary Table 6: Mixed effects model measuring the cross-cluster surprisal effect including the first 500 trials. A mixed effects model fit to all of the reaction time data, including the first 500 trials for each subject, for the modular graph with the goal of measuring the cross-cluster surprisal effect. We find a significant 35 ms increase in reaction times (173 subjects) for between-cluster transitions versus within-cluster transitions. The significance column represents p-values less than 0.001 ($***$), less than 0.01 ($**$), and less than 0.05 ($*$). Source data are provided as a Source Data file.

in the previous analysis in Sec. 3 (i.e., 'RT $\sim \log(\text{Trial}) * \text{Stage} + \text{Target} + \text{Recency} + \text{Graph} + (1 + \log(\text{Trial}) * \text{Stage} + \text{Recency} \,|\, \text{ID})$'), and including all 1500 trials for each subject, we find a significant 16 ms increase in reaction times in the lattice versus the modular graph (Supplementary Table 7). These results demonstrate that higher-order network effects studied in the main text exist throughout the entire serial response task.

Effect	Estimate (ms)	t-value	Pr(>|t|)	Significance
(Intercept)	1357.0 ± 30.3	44.79	< 0.001	$* * *$
$\log(\text{Trial})$	-87.8 ± 3.4	-26.06	< 0.001	$* * *$
Stage	-490.7 ± 25.3	-19.38	< 0.001	$* * *$
Recency	2.0 ± 0.1	32.35	< 0.001	$* * *$
Graph	16.3 ± 5.4	3.00	0.003	$**$
$\log(\text{Trial})$:Stage	62.7 ± 3.5	17.76	< 0.001	$* * *$

Supplementary Table 7: Mixed effects model measuring the modular-lattice effect including the first 500 trials. A mixed effects model fit to all of the reaction time data, including the first 500 trials for each subject, for the modular and lattice graphs with the goal of measuring the modular-lattice effect. We find a significant 16 ms increase in reaction times overall (72 subjects) in the lattice graph relative to the modular graph. The significance column represents p-values less than 0.001 ($* * *$), less than 0.01 ($**$), and less than 0.05 ($*$). Source data are provided as a Source Data file.

7 Network effects on error trials

Thus far we have focused on predicting human reaction times as a proxy for people's anticipations of transitions. Another way to probe anticipation is by studying the trials on which subjects respond incorrectly; one might expect that the probability of an erroneous response should increase with decreasing anticipation. Here, we test this hypothesis for between- versus within-cluster transitions in the modular graph and for all transitions in the modular graph versus the lattice graph.

7.1 Cross-cluster surprisal effect on errors

First, we consider the cross-cluster surprisal effect on errors defined by an increase in task errors for transitions between clusters relative to transitions within clusters in the modular graph. We employ a mixed effects model with formula 'Error $\sim \log(\text{Trial})$ + Stage + Target + Recency + Trans_Type + $(1 + \log(\text{Trial})\,|\,\text{ID})$', where Error indicates whether the subject provided an incorrect ('1') or correct ('0') response. Note that, relative to our measurement of the cross-cluster surprisal for reaction times in Sec. 3, we have removed the fixed effect interaction between $\log(\text{Trial})$ and Stage as well as the mixed effects for the variables Stage, Recency, and Trans_Type because they are not statistically significant in this setting. We find a significant increase in errors for between- versus within-cluster transitions (Supplementary Table 8), suggesting yet again that subjects have weaker anticipation for cross-cluster transitions than for within-cluster transitions.

7.2 Modular-lattice effect on errors

Second, we consider the modular-lattice effect on errors defined by an increase in task errors for the lattice graph relative to the modular graph. We employ a mixed effects model with formula 'Error $\sim \log(\text{Trial})$ + Stage + Target + Recency + Graph + $(1 + \log(\text{Trial})$ + Recency + Graph | ID)', where each of the variables has been defined previously. We again note that we have removed the interaction between $\log(\text{Trial})$ and Stage because it was not statistically significant in our prediction of task errors. Inspecting the mixed effects model described in Supplementary Table 9, we do not find a significant difference in the number of task errors between the modular and lattice graphs. One possible explanation for this lack of an effect is that people's task accuracy is predominantly

Effect	Estimate	t-value	Pr(>|t|)	Significance
(Intercept)	0.005 ± 0.012	0.39	0.697	
$\log$(Trial)	0.004 ± 0.002	2.14	0.032	$*$
Stage	0.015 ± 0.007	2.14	0.032	$*$
Recency	< 0.001	16.52	< 0.001	$* * *$
Trans_Type	0.004 ± 0.002	2.83	0.005	$**$

Supplementary Table 8: Mixed effects model measuring the cross-cluster effect on task errors. A mixed effects model fit to predict error trials for the modular graph with the goal of measuring the cross-cluster effect on task errors. We find a significant increase in task errors (173 subjects) for between-cluster transitions relative to within-cluster transitions (grey). The significance column represents p-values less than 0.001 ($* * *$), less than 0.01 ($**$), and less than 0.05 ($*$). Source data are provided as a Source Data file.

impacted by very poorly anticipated transitions. Thus, while anticipation in the lattice graph is lower than that in the modular graph on average, it could be the case that the significant decrease in anticipation for cross-cluster transitions in the modular graph yields a similar number of task errors overall.

8 Measuring the effects of network violations

We study the effects of violations of varying topological distance in the ring graph using a mixed effects model with the formula 'RT $\sim \log(\text{Trial}) + \text{Target} + \text{Recency} + \text{Top\_Dist} + (1 + \log(\text{Trial}) + \text{Recency} + \text{Top\_Dist} \,|\, \text{ID})$', where Top_Dist represents the topological distance of a transition, either one for a standard transition, two for a short violation, or three for a long violation. The results

Effect	Estimate	t-value	Pr(>|t|)	Significance
(Intercept)	0.026 ± 0.009	3.05	0.002	$**$
$\log$(Trial)	0.002 ± 0.001	1.47	0.142	
Stage	0.003 ± 0.003	0.98	0.325	
Recency	< 0.001	14.62	< 0.001	$***$
Graph	-0.004 ± 0.003	-1.34	0.180	

Supplementary Table 9: Mixed effects model measuring the modular-lattice effect on task errors. A mixed effects model fit to predict error trials for the modular and lattice graphs with the goal of measuring the modular-lattice effect on task errors. We do not find a significant change in errors based on the graph (grey; 72 subjects). The significance column represents p-values less than 0.001 ($***$), less than 0.01 ($**$), and less than 0.05 ($*$). Source data are provided as a Source Data file.

of fitting this mixed effects model are summarized in Supplementary Table 10, reporting increases in reaction times over standard transitions of 38 ms for short violations and 63 ms for long violations. Second, to measure the difference in reaction times between long and short violations, we implemented a model of the same form, but restricted Top_Dist to only include short violations of topological distance two and long violations of topological distances three and four. This model is summarized in Supplementary Table 11, reporting a 28 ms increase in reaction times for long violations relative to short violations. This result is measured from all 78 subjects that observed random walks with violations in the ring graph.

Effect	Estimate (ms)	t-value	Pr(>|t|)	Significance
(Intercept)	1352.7 ± 79.2	17.07	< 0.001	$***$
$\log$(Trial)	-101.1 ± 10.2	-9.96	< 0.001	$***$
Recency	2.1 ± 0.1	16.20	< 0.001	$***$
Top_Dist (short vs. no violation)	37.9 ± 8.4	4.50	< 0.001	$***$
Top_Dist (long vs. no violation)	63.3 ± 7.8	8.07	< 0.001	$***$

Supplementary Table 10: Mixed effects model measuring the effects of violations relative to standard transitions. A mixed effects model fit to the reaction time data for the ring graph with the goal of measuring the effects of violations relative to standard transitions. We find a significant increase in reaction times of 38 ms (78 subjects) for short violations and 63 ms for long violations (grey), even after accounting for recency effects. The significance column represents p-values less than 0.001 ($***$), less than 0.01 ($**$), and less than 0.05 ($*$). Source data are provided as a Source Data file.

9 Controlling for recency: Network violations

In the main text, we attribute the observed increase in reaction times for network violations to subjects' internal representations of the transition structure. Alternatively, these effects could be due to the fact that standard transitions are more likely than network violations to yield a stimulus that has been seen recently. To show that the effects of network violations are not simply driven by recency, we directly control for the recency of stimuli in our data. Because the violations data is more sparse than the standard random walk data (we only observe 50 violations per subject, split between 20 short violations and 30 long violations), and because the network violations often yield stimuli with large recency values (for example, 69% of violations yield stimuli with recency greater

Effect	Estimate (ms)	t-value	Pr(>|t|)	Significance
(Intercept)	1380.9 ± 156.1	8.84	< 0.001	$***$
$\log$(Trial)	-97.1 ± 21.3	-4.57	< 0.001	$***$
Recency	0.7 ± 0.3	2.67	0.008	$**$
Top_Dist (long vs. short violation)	28.4 ± 11.2	2.54	0.011	$*$

Supplementary Table 11: Mixed effects model measuring the effects of long versus short violations. A mixed effects model fit to the reaction time data for the ring graph with the goal of measuring the effects of long versus short violations. We find a significant 28 ms increase in reaction times (78 subjects) for long violations relative to short violations (grey), even after accounting for recency effects. The significance column represents p-values less than 0.001 ($***$), less than 0.01 ($**$), and less than 0.05 ($*$). Source data are provided as a Source Data file.

than 10), we separate our data based on ranges of recency values that provide an approximately even distribution of violations (see Supplementary Fig. 4a). After separating the data by recency, we estimate the effects of short and long violations using a mixed effects model of the form 'RT $\sim$ $\log$(Trial) + Target + Top_Dist + $(1 + \log(\text{Trial}) \mid \text{ID})$'. We note that, in comparison to the model used in Sec. 8, we have removed the mixed effect of Top_Dist because the filtered datasets are not large enough to provide a significant estimate. In Supplementary Fig. 4a, we see that, within each recency range other than recency greater than 40, at least one of either the short or long violations generates a significant increase in reaction times relative to standard transitions. Additionally, in Supplementary Fig. 4b, we filter the violations data by the number of appearances of the current stimulus in the previous 10 trials. Network violations yield significant increases in reaction times

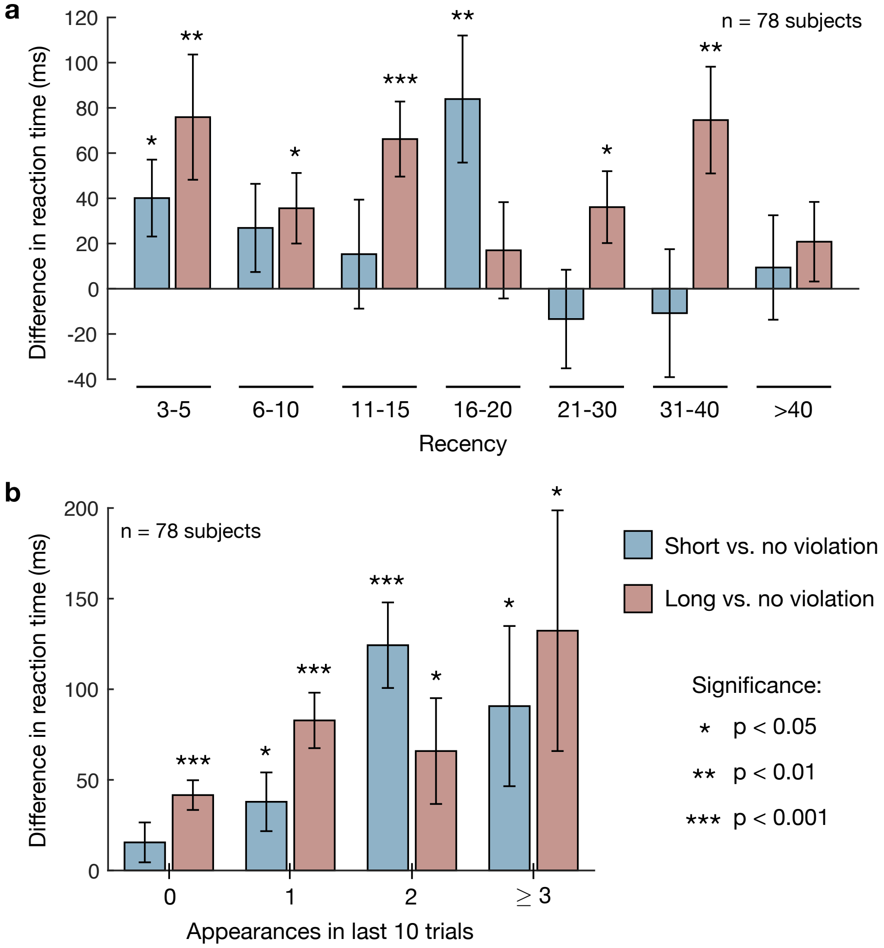

Supplementary Fig. 4: Comparing standard transitions to network violations while controlling for recency.

a, Difference in reaction times between standard transitions and short violations (blue) or long violations (red) in the ring graph after controlling for the recency of stimuli. We observe at least one significant effect of network violations for all recency ranges less than 40. **b**, Increase in reaction times for short (blue) and long (red) network violations after controlling for the number of times the current stimulus has appeared in the previous 10 trials. For long violations, we find a significant increase in reaction times across all numbers of recent stimulus appearances. For short violations, we find a significant increase in reaction times across all numbers of recent stimulus appearances besides zero. Effect sizes (represented by bar plots), standard deviations (represented by error bars), and p-values are estimated using mixed effects models. The results are measured for all 78 subjects that observed random walks with violations in the ring graph. Source data are provided as a Source Data file.

across all conditions other than short violations with zero appearances in the last 10 trials. Together, these results demonstrate that the effects of network violations cannot simply be explained by recency, therefore suggesting that subjects maintain an internal representation of the transition structure.

We repeat the above analysis to measure the difference in reaction times between short and long violations while controlling for recency. We observe a significant increase in reaction times for long violations relative to short violations in two of the seven recency ranges (Supplementary Fig. 5a). However, we do not report a significant difference in reaction times after controlling for the number of appearances of stimuli in the previous 10 trials (Supplementary Fig. 5b). We remark that, given the noisy nature of reaction times and the small number of measurements per subject, the large standard deviations in our estimates are not surprising. Nevertheless, these results provide tentative evidence that recency alone cannot explain the difference in reaction times between long and short network violations.

10 The forgetting of stimuli cannot explain network effects

In the derivation of our model, the central mathematical object is the memory distribution $P(\Delta t)$, which represents the probability that a person recalls the stimulus that occurred at time $t - \Delta t$ instead of the stimulus that they were trying to recall at time t. Generally, this memory distribution is intended to reflect the erroneous shuffling of past stimuli in a person's memory. Alternatively, one could imagine errors in memory that reflect the forgetting of past stimuli altogether, a process that has recently been shown to impact human reinforcement learning[9,10] and to facilitate flexible

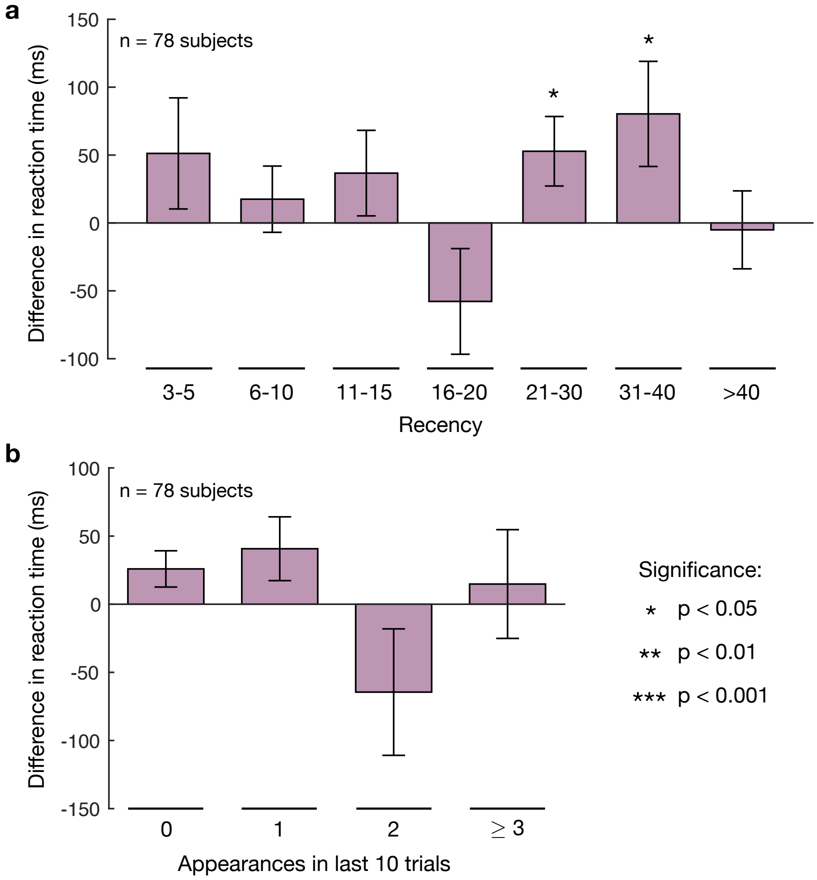

Supplementary Fig. 5: Comparing short versus long network violations while controlling for recency.

a, Difference in reaction times between short and long network violations after controlling for the recency of stimuli. We find significant increases in reaction times for long violations in the recency ranges 21-30 and 31-40. **b**, Difference in reaction times between short and long network violations after controlling for the number of times the current stimulus has appeared in the previous 10 trials. Effect sizes (represented by bar plots), standard deviations (represented by error bars), and *p*-values are estimated using mixed effects models. The results are measured for all 78 subjects that observed random walks with violations in the ring graph. Source data are provided as a Source Data file.

and generalizable decision making.[11] Here we argue that this second form of cognitive errors – that is, the simple forgetting of stimuli – cannot explain the higher-order network effects described in the main text.

Consider a sequence of stimuli reflecting a random walk of length T on a network defined by the transition matrix A, where A_{ij} represents the probability of transitioning from stimulus i to stimulus j. Given a running tally $n_{ij}(T)$ of the number of times each transition has occurred, we recall that the most accurate prediction for the transition structure is given by the maximum likelihood estimate $\hat{A}_{ij}^{\text{MLE}}(T) = n_{ij}(T)/\sum_k n_{ik}(T)$. Now suppose that a human learner forgets each observed transition at some fixed rate. On average, this process of estimating A after forgetting some number of transitions uniformly at random is equivalent to estimating A at some prior time T_{eff}. In other words, forgetting observed transitions at random simply introduces additional white noise into the transitions estimates $\hat{A}_{ij}^{\text{MLE}}(T)$. As discussed in the main text, maximum likelihood estimation provides an unbiased estimate of the transition structure, and therefore cannot explain the fact that people's representations depend systematically on higher-order network organization. Similarly, the addition of white noise to $\hat{A}^{\text{MLE}}(T)$ will also yield an unbiased (although less accurate) estimate of the transition structure. Therefore, while the forgetting of past stimuli plays an important role in a number of cognitive processes,[9–11] this mechanism cannot be used to explain the higher-order network effects observed in human experiments and predicted by our model.

11 Gradient of RMS error with respect to inverse temperature β

Given a sequence of nodes x_t, we recall that our prediction for the reaction time at time t is given by $\hat{r}(t) = r_0 + r_1 a(t)$, where $a(t) = \hat{A}_{x_{t-1},x_t}(t-1)$ is the predicted anticipation of node x_t. The gradient of the RMS error $\text{RMSE} = \sqrt{\frac{1}{T}\sum_t (r(t) - \hat{r}(t))^2}$ with respect to the inverse temperature

β is given by

$$\frac{\partial \text{RMSE}}{\partial \beta} = \frac{-r_1}{T}\frac{1}{\text{RMSE}}\sum_t \left(r(t) - \hat{r}(t)\right)\frac{\partial a(t)}{\partial \beta}, \tag{S1}$$

where the derivative of the anticipation is given by

$$\frac{\partial \hat{A}_{ij}(t)}{\partial \beta} = \frac{1}{\sum_k \tilde{n}_{ik}(t)}\left(\frac{\partial \tilde{n}_{ij}(t)}{\partial \beta} - \hat{A}_{ij}(t)\sum_\ell \frac{\partial \tilde{n}_{i\ell}(t)}{\partial \beta}\right). \tag{S2}$$

Recalling Eq. (8) from the main text, the derivative of the transition counts can be written

$$\frac{\partial \tilde{n}_{ij}(t)}{\partial \beta} = \sum_{t'=1}^{t-1}\sum_{\Delta t=0}^{t'-1}\frac{\partial P_{t'}(\Delta t)}{\partial \beta}\left[i = x_{t'-\Delta t}\right]\left[j = x_{t'+1}\right], \tag{S3}$$

where $P_{t'}(\Delta t)$ represents the probability of accidentally remembering the node $x_{t'-\Delta t}$ instead of the target node $x_{t'}$. Taking one more derivative, we have

$$\frac{\partial P_{t'}(\Delta t)}{\partial \beta} = P_{t'}(\Delta t)\left(-\Delta t + \sum_{\Delta t'=0}^{t'-1} P_{t'}(\Delta t')\Delta t'\right). \tag{S4}$$

Taken together, Eqs. (S1)-(S4) define the derivative of the RMS error with respect to the inverse temperature β, thus completing the description of our gradient descent algorithm.

12 Connection to the successor representation

In the limit of an infinitely-long sequence of nodes, we showed in the main text that the transition estimates in our model take the following concise analytic form,

$$\hat{A} = (1 - e^{-\beta})A(I - e^{-\beta}A)^{-1}, \tag{S5}$$

where A is the true transition structure, β is the inverse temperature in our memory distribution, and I is the identity matrix. Interestingly, this equation takes a similar form to the successor

representation from reinforcement learning,

$$M = A(I - \gamma A)^{-1}, \tag{S6}$$

where γ is the future discount factor, which tunes the desired time-scale over which a person wishes to make predictions.[12,13] Put simply, starting at some node i, the successor representation M_{ij} counts the future discounted occupancy of node j. Identifying $\gamma = e^{-\beta}$, we notice that the successor representation is equivalent to an unnormalized version of our transition estimates. Moreover, the same mathematical form crops up in complex network theory, where it is known as the communicability between nodes in a graph.[14–16]

The relationship between the transition estimates in our model and the successor representation is fascinating, especially given the marked differences in the concepts that the two models are based upon. In our model, people attempting to learn the one-step transition structure A instead arrive at the erroneous estimate $\hat{A}$ due to natural errors in perception and recall. By contrast, given a desired time-scale γ, the successor representation defines the optimal prediction of node occupancies into the future.[12,13] Interestingly, the successor representation has been linked to grid cells and abstract representations in the hippocampus,[16,17] decision making in reward-based tasks,[18,19] and the temporal difference and temporal context models of learning and memory.[12,13,20] The successor representation assumes that humans are making predictions multiple steps into the future; however, our results show that a similar mathematical form can instead represent a person who simply attempts to predict one step into the future, but misses the mark due to natural errors in cognition. This biologically-plausible hypothesis of erroneous predictions highlights the importance of thinking carefully about the impact of mental errors on human learning.[9–11]

References

1. Fiser, J. & Aslin, R. N. Statistical learning of higher-order temporal structure from visual shape sequences. *J. Exp. Psychol.* **28**, 458 (2002).

2. Kahn, A. E., Karuza, E. A., Vettel, J. M. & Bassett, D. S. Network constraints on learnability of probabilistic motor sequences. *Nat. Hum. Behav.* **2**, 936 (2018).

3. Saffran, J. R., Aslin, R. N. & Newport, E. L. Statistical learning by 8-month-old infants. *Science* **274**, 1926–1928 (1996).

4. Schall, R. Estimation in generalized linear models with random effects. *Biometrika* **78**, 719–727 (1991).

5. Baayen, R. H., Davidson, D. J. & Bates, D. M. Mixed-effects modeling with crossed random effects for subjects and items. *J. Mem. Lang.* **59**, 390–412 (2008).

6. Bates, D., Mächler, M., Bolker, B., Walker, S. *et al.* Fitting linear mixed-effects models using lme4. *J. Stat. Softw.* **67** (2015).

7. Baddeley, A. D. & Hitch, G. The recency effect: Implicit learning with explicit retrieval? *Mem. Cogn.* **21**, 146–155 (1993).

8. Schapiro, A. C., Rogers, T. T., Cordova, N. I., Turk-Browne, N. B. & Botvinick, M. M. Neural representations of events arise from temporal community structure. *Nat. Neurosci.* **16**, 486–492 (2013).

9. Collins, A. G. & Frank, M. J. How much of reinforcement learning is working memory, not reinforcement learning? A behavioral, computational, and neurogenetic analysis. *Eur. J. Neurosci.* **35**, 1024–1035 (2012).

10. Collins, A. G. & Frank, M. J. Within-and across-trial dynamics of human EEG reveal cooperative interplay between reinforcement learning and working memory. *Proc. Natl. Acad. Sci. U.S.A.* 201720963 (2018).

11. Richards, B. A. & Frankland, P. W. The persistence and transience of memory. *Neuron* **94**, 1071–1084 (2017).

12. Sutton, R. S. Td models: Modeling the world at a mixture of time scales. In *Machine Learning Proceedings 1995*, 531–539 (Elsevier, 1995).

13. Gershman, S. J., Moore, C. D., Todd, M. T., Norman, K. A. & Sederberg, P. B. The successor representation and temporal context. *Neural Comput.* **24**, 1553–1568 (2012).

14. Estrada, E. & Hatano, N. Communicability in complex networks. *Phys. Rev. E* **77**, 036111 (2008).

15. Estrada, E., Hatano, N. & Benzi, M. The physics of communicability in complex networks. *Phys. Rep.* **514**, 89–119 (2012).

16. Garvert, M. M., Dolan, R. J. & Behrens, T. E. A map of abstract relational knowledge in the human hippocampal–entorhinal cortex. *Elife* **6** (2017).

17. Stachenfeld, K. L., Botvinick, M. M. & Gershman, S. J. The hippocampus as a predictive map. *Nat. Neurosci.* **20**, 1643 (2017).

18. Momennejad, I. *et al.* The successor representation in human reinforcement learning. *Nat. Hum. Behav.* **1**, 680 (2017).

19. Russek, E. M., Momennejad, I., Botvinick, M. M., Gershman, S. J. & Daw, N. D. Predictive representations can link model-based reinforcement learning to model-free mechanisms. *PLOS Comput. Biol.* **13**, e1005768 (2017).

20. Howard, M. W. & Kahana, M. J. A distributed representation of temporal context. *J. Math. Psychol.* **46**, 269–299 (2002).
