# Supplementary material for: Abstract representations of events arise from mental errors in learning and memory
Source: Nat Commun. 2020 May 8;11:2313. doi: 10.1038/s41467-020-15146-7 (PMC7210268; doi:10.1038/s41467-020-15146-7)
Supplement: Supplementary file 2 — Reporting Summary [file 41467_2020_15146_MOESM2_ESM.pdf]

nature research

Corresponding author(s): Danielle S. Bassett

Last updated by author(s): 2/5/2020

Reporting Summary

Nature Research wishes to improve the reproducibility of the work that we publish. This form provides structure for consistency and transparency in reporting. For further information on Nature Research policies, see Authors & Referees and the Editorial Policy Checklist.

Statistics

For all statistical analyses, confirm that the following items are present in the figure legend, table legend, main text, or Methods section.

n/a	Confirmed	
☐	☒	The exact sample size (n) for each experimental group/condition, given as a discrete number and unit of measurement
☐	☒	A statement on whether measurements were taken from distinct samples or whether the same sample was measured repeatedly
☐	☒	The statistical test(s) used AND whether they are one- or two-sided<br>*Only common tests should be described solely by name; describe more complex techniques in the Methods section.*
☐	☒	A description of all covariates tested
☐	☒	A description of any assumptions or corrections, such as tests of normality and adjustment for multiple comparisons
☐	☒	A full description of the statistical parameters including central tendency (e.g. means) or other basic estimates (e.g. regression coefficient) AND variation (e.g. standard deviation) or associated estimates of uncertainty (e.g. confidence intervals)
☐	☒	For null hypothesis testing, the test statistic (e.g. F, t, r) with confidence intervals, effect sizes, degrees of freedom and P value noted<br>*Give P values as exact values whenever suitable.*
☐	☒	For Bayesian analysis, information on the choice of priors and Markov chain Monte Carlo settings
☐	☒	For hierarchical and complex designs, identification of the appropriate level for tests and full reporting of outcomes
☐	☒	Estimates of effect sizes (e.g. Cohen's d, Pearson's r), indicating how they were calculated

Our web collection on statistics for biologists contains articles on many of the points above.

Software and code

Policy information about availability of computer code

Data collection	Custom code was used to collect data on Amazon's Mechanical Turk. This code has been made publicly available, and can be found at https://github.com/ariekahn/psiTurk.
Data analysis	To analyze data, we use built-in functions in MATLAB (R2018a).

For manuscripts utilizing custom algorithms or software that are central to the research but not yet described in published literature, software must be made available to editors/reviewers. We strongly encourage code deposition in a community repository (e.g. GitHub). See the Nature Research guidelines for submitting code & software for further information.

Data

Policy information about availability of data

All manuscripts must include a data availability statement. This statement should provide the following information, where applicable:

- Accession codes, unique identifiers, or web links for publicly available datasets
- A list of figures that have associated raw data
- A description of any restrictions on data availability

Source data for Fig. \ref{experiment} are provided in Supplementary Data File 1. Source data for Fig. \ref{effects}, Supplementary Figs. 2 and 3, and Supplementary Tables 1-9 are provided in Supplementary Data File 2. Source data for Fig. \ref{nback} are provided in Supplementary Data File 3. Source data for Fig. \ref{violations}, Supplementary Figs. 4 and 5, and Supplementary Tables 10 and 11 are provided in Supplementary Data File 4. Source data from Supplementary Fig. 1 are provided in Supplementary Data File 5.

Field-specific reporting

Please select the one below that is the best fit for your research. If you are not sure, read the appropriate sections before making your selection.

☐ Life sciences ☒ Behavioural & social sciences ☐ Ecological, evolutionary & environmental sciences

For a reference copy of the document with all sections, see nature.com/documents/nr-reporting-summary-flat.pdf

Behavioural & social sciences study design

All studies must disclose on these points even when the disclosure is negative.

Study description	We perform two types of studies, both quantitative in nature. In the first study type, we measure participants' reaction times and error rates in serial response experiments. In the second study type, we record participants' responses in a standard n-back working memory experiment.
Research sample	Participants were selected using Amazon's Mechanical Turk, which provides unprecedented access to human behavioral data from participants around the globe. Using this population allowed us to gather large amounts of behavioral data from a multi-national sample of participants. Previously-collected data, also from Amazon's Mechanical Turk, was used in some of our analyses; this data can be found in [Kahn, Ari E., et al., Nat. Hum. Behav. (2018)].
Sampling strategy	Participants signed up for the experiments voluntarily online. We ensured that each participant was unique to preclude the possibility of a participant performing two different experiments. We selected sample sizes based on previously-used protocols described in [Kahn, Ari E., et al., Nat. Hum. Behav. (2018)].
Data collection	The data was collected on Amazon's Mechanical Turk. The data were collected on participants' personal computers with no researchers present.
Timing	Data was originally collected between 3/26/18 and 5/1/18. Through multiple rounds of revisions, we collected additional data from 10/4/18 to 6/29/19.
Data exclusions	No subjects were excluded from analyses. We excluded all trials in which subjects responded incorrectly. Finally, we excluded reaction times that were implausible, either three standard deviations from a subject's mean reaction time, below 100 ms, or over 3500 ms.
Non-participation	No participants dropped out or declined participation.
Randomization	Participants were not allocated into experimental groups.

Reporting for specific materials, systems and methods

We require information from authors about some types of materials, experimental systems and methods used in many studies. Here, indicate whether each material, system or method listed is relevant to your study. If you are not sure if a list item applies to your research, read the appropriate section before selecting a response.

Materials & experimental systems

n/a	Involved in the study	
☒	☐	Antibodies
☒	☐	Eukaryotic cell lines
☒	☐	Palaeontology
☒	☐	Animals and other organisms
☐	☒	Human research participants
☒	☐	Clinical data

Methods

n/a	Involved in the study	
☒	☐	ChIP-seq
☒	☐	Flow cytometry
☒	☐	MRI-based neuroimaging

Human research participants

Policy information about studies involving human research participants

Population characteristics	See above.
Recruitment	Participants signed up for the experiments voluntarily on Amazon's Mechanical Turk. Given the simplicity of the experiments, we do not foresee any biases that would reflect the results.
Ethics oversight	All participants provided informed consent in writing and experimental methods were approved by the Institutional Review Board of the University of Pennsylvania.

Note that full information on the approval of the study protocol must also be provided in the manuscript.
